# Supplementary material for: Bactericidal effect of far ultraviolet-C irradiation at 222 nm against bacterial peritonitis
Source: PLoS One. 2024 Nov 12;19(11):e0311552. doi: 10.1371/journal.pone.0311552 (PMC11556692; doi:10.1371/journal.pone.0311552)

PLOS ONE

Bactericidal effect of far ultraviolet-C irradiation at 222 nm against bacterial peritonitis

--Manuscript Draft--

**Manuscript Number:**	PONE-D-24-00293
**Article Type:**	Research Article
**Full Title:**	Bactericidal effect of far ultraviolet-C irradiation at 222 nm against bacterial peritonitis
**Short Title:**	Short title: Far UV-C against bacterial peritonitis
**Corresponding Author:**	Kiyotaka Kurachi, MD.PhD.<br>Hamamatsu University School of Medicine: Hamamatsu Ika Daigaku<br>Hamamatsu, Shizuoka JAPAN
**Keywords:**	222 nm UV-C; Far UV-C; UV-C; peritonitis; bacterial peritonitis; E-coli; lavage; surgery; surgical treatment
**Abstract:**	Far ultraviolet-C irradiation at a wavelength of 222 nm has strong bactericidal effects against severe infections, such as peritonitis, but is rarely cytotoxic. Bacterial peritonitis due to bowel perforation is a severe disease with a high mortality rate despite having available treatment options. The effects of intraperitoneal far ultraviolet-C irradiation at 222 nm are unknown; therefore, herein, we investigated its safety and efficacy. We performed in vitro experiments to optimize the fluid conditions of bacterial or protein concentration for the in vivo evaluation of the bactericidal effect. We evaluated the efficacy of far ultraviolet-C irradiation at 222 nm during lavage of Escherichia coli-induced peritonitis rats by analyses of the intra-abdominal bacteria concentrations, blood cytokine concentrations, and mortality. We also evaluated the safety of far ultraviolet-C irradiation at 222 nm by evaluation of cyclobutane pyrimidine dimers as DNA damage in five abdominal organs, namely the stomach, small intestine, colon, liver, and spleen. Statistical analyses were performed using parametric analysis in the data with normality, and using non-parametric analysis in the data without normality. The results of the in vitro experiments revealed the optimal conditions is, 106 CFU/mL of bacteria, 0.5 mW/cm2 of irradiation, and 10-3 mg/mL of protein. In the rat peritonitis model, far ultraviolet-C irradiation at 222 nm reduced the intra-abdominal bacteria and blood cytokine (interleukin-1β and interleukin-6) concentrations and improved mortality (from 20% to 60%) compared with only lavage. Cyclobutane pyrimidine dimer formation was less observed in organ tissues with far ultraviolet-C irradiation at 222 nm than in those with ultraviolet-C irradiation at 254 nm. Our findings suggest far ultraviolet-C irradiation at 222 nm during lavage as a therapeutic against bacterial peritonitis, with a good safety profile.
**Order of Authors:**	Kosuke Sugiyama
	Kiyotaka Kurachi
	Masaki Sano
	Kyota Tatsuta
	Tadahiro Kojima
	Toshiya Akai
	Katsunori Suzuki
	Kakeru Torii
	Mayu Sakata
	Yoshifumi Morita
	Hirotoshi Kikuchi
	Yoshihiro Hiramatsu
	Yohei Kumabe
	Keisuke Oe

Powered by Editorial Manager® and ProduXion Manager® from Aries Systems Corporation

	Tomoaki Fukui
	Rena Kaigome
	Masahiro Sasaki
	Toru Koi
	Hiroyuki Ohashi
	Tetsuro Suzuki
	Ryosuke Kuroda
	Hiroya Takeuchi
**Additional Information:**	
**Question**	**Response**
**Financial Disclosure**<br><br>Enter a financial disclosure statement that describes the sources of funding for the work included in this submission. Review the submission guidelines for detailed requirements. View published research articles from *PLOS ONE* for specific examples.<br><br>This statement is required for submission and **will appear in the published article** if the submission is accepted. Please make sure it is accurate.<br><br>**Funded studies**<br>Enter a statement with the following details:<br>• Initials of the authors who received each award<br>• Grant numbers awarded to each author<br>• The full name of each funder<br>• URL of each funder website<br>• Did the sponsors or funders play any role in the study design, data collection and analysis, decision to publish, or preparation of the manuscript?<br><br>Did you receive funding for this work?	The author(s) received no specific funding for this work.
**Competing Interests**<br><br>Use the instructions below to enter a competing interest statement for this submission. On behalf of all authors, disclose any competing interests that could be perceived to bias this	Because this research required specialized experimental equipment, it was started as a joint research project between Kobe University, Ushio Inc., and Hamamatsu University of Medicine. This experiment was performed at Hamamatsu University School of Medicine. The staff of Ushio Inc. and Kobe University lent specialized equipment used in another study to the Hamamatsu University School of Medicine, evaluated its appropriate use, assessed the validity of the methods and results, and confirmed the reproducibility of some data. Thus, the funders had no role in data

work—acknowledging all financial support and any other relevant financial or non-financial competing interests.<br><br>This statement is required for submission and **will appear in the published article** if the submission is accepted. Please make sure it is accurate and that any funding sources listed in your Funding Information later in the submission form are also declared in your Financial Disclosure statement.<br><br>View published research articles from *PLOS ONE* for specific examples.<br><br>**NO authors have competing interests**<br><br>Enter: *The authors have declared that no competing interests exist.*<br><br>**Authors with competing interests**<br><br>Enter competing interest details beginning with this statement:<br><br>*I have read the journal's policy and the authors of this manuscript have the following competing interests: [insert competing interests here]*<br><br>* typeset	collection and analysis. Based on the result of this research, Hamamatsu University School of Medicine have received funds from Ushio Inc. associated with this collaborative project. Kobe University School of Medicine and Hamamatsu University School of Medicine are funded by Ushio Inc. for collaborating research. RK, MS, TK, and HO have received support in the form of salaries from Ushio Inc.
**Ethics Statement**<br><br>Enter an ethics statement for this submission. This statement is required if the study involved:<br><br>• Human participants<br>• Human specimens or tissue<br>• Vertebrate animals or cephalopods<br>• Vertebrate embryos or tissues<br>• Field research<br><br>Write "N/A" if the submission does not require an ethics statement.	All animal procedures and experimental protocols were performed in accordance with the ethical standards of the Animal Care and Use Committee of the Hamamatsu University School of Medicine (approval number 2022008). The study protocols complied with the Animal Research Reporting of In Vivo Experiments guidelines.<br>All rats were intraperitoneally anesthetized with 0.4 mg/kg medetomidine, 2.0 mg/kg midazolam, and 5.0 mg/kg butorphanol before the surgical procedures. Euthanasia was performed with an overdose of intraperitoneal anesthetics (1.2 mg/kg medetomidine, 6.0 mg/kg midazolam, and 15.0 mg/kg butorphanol).

Powered by Editorial Manager® and ProduXion Manager® from Aries Systems Corporation

General guidance is provided below. Consult the submission guidelines for detailed instructions. **Make sure that all information entered here is included in the Methods section of the manuscript.**<br><br>**Format for specific study types**<br><br>**Human Subject Research (involving human participants and/or tissue)**<br>• Give the name of the institutional review board or ethics committee that approved the study<br>• Include the approval number and/or a statement indicating approval of this research<br>• Indicate the form of consent obtained (written/oral) or the reason that consent was not obtained (e.g. the data were analyzed anonymously)<br><br>**Animal Research (involving vertebrate animals, embryos or tissues)**<br>• Provide the name of the Institutional Animal Care and Use Committee (IACUC) or other relevant ethics board that reviewed the study protocol, and indicate whether they approved this research or granted a formal waiver of ethical approval<br>• Include an approval number if one was obtained<br>• If the study involved *non-human primates*, add *additional details* about animal welfare and steps taken to ameliorate suffering<br>• If anesthesia, euthanasia, or any kind of animal sacrifice is part of the study, include briefly which substances and/or methods were applied<br><br>**Field Research**<br><br>Include the following details if this study involves the collection of plant, animal, or other materials from a natural setting:<br>• Field permit number<br>• Name of the institution or relevant body that granted permission	
**Data Availability**<br><br>Authors are required to make all data underlying the findings described fully	Yes - all data are fully available without restriction

Powered by Editorial Manager® and ProduXion Manager® from Aries Systems Corporation

available, without restriction, and from the time of publication. PLOS allows rare exceptions to address legal and ethical concerns. See the PLOS Data Policy and FAQ for detailed information.<br><br>A Data Availability Statement describing where the data can be found is required at submission. Your answers to this question constitute the Data Availability Statement and **will be published in the article**, if accepted.<br><br>**Important:** Stating 'data available on request from the author' is not sufficient. If your data are only available upon request, select 'No' for the first question and explain your exceptional situation in the text box.<br><br>Do the authors confirm that all data underlying the findings described in their manuscript are fully available without restriction?	
**Describe where the data may be found in full sentences. If you are copying our sample text, replace any instances of XXX with the appropriate details.**<br><br>• If the data are **held or will be held in a public repository**, include URLs, accession numbers or DOIs. If this information will only be available after acceptance, indicate this by ticking the box below. For example: *All XXX files are available from the XXX database (accession number(s) XXX, XXX.*).<br>• If the data are all contained **within the manuscript and/or Supporting Information files**, enter the following: *All relevant data are within the manuscript and its Supporting Information files.*<br>• If neither of these applies but you are able to provide **details of access elsewhere**, with or without limitations, please do so. For example:<br><br>*Data cannot be shared publicly because of [XXX]. Data are available from the XXX Institutional Data Access / Ethics*	All relevant data are within the manuscript and its Supporting Information files.

Committee (contact via XXX) for researchers who meet the criteria for access to confidential data.<br><br>*The data underlying the results presented in the study are available from (include the name of the third party and contact information or URL).*<br>• This text is appropriate if the data are owned by a third party and authors do not have permission to share the data.<br><br>* typeset	
Additional data availability information:	

Powered by Editorial Manager® and ProduXion Manager® from Aries Systems Corporation

Manuscript
Click here to access/download;Manuscript;222nm UV-C manuscript.docx

Bactericidal effect of far ultraviolet-C irradiation at 222 nm against bacterial peritonitis

Short title: Far UV-C against bacterial peritonitis

Kosuke Sugiyama[1], Kiyotaka Kurachi[1]*, Masaki Sano[1], Kyota Tatsuta[1], Tadahiro Kojima[1], Toshiya Akai[1], Katsunori Suzuki[1], Kakeru Torii[1], Mayu Sakata[1], Yoshifumi Morita[1], Hirotoshi Kikuchi[1], Yoshihiro Hiramatsu[1,5], Yohei Kumabe[2], Keisuke Oe[2], Tomoaki Fukui[2], Rena Kaigome[3], Masahiro Sasaki[3], Toru Koi[3], Hiroyuki Ohashi[3], Tetsuro Suzuki[4], Ryosuke Kuroda[2], Hiroya Takeuchi[1]

[1] Department of Surgery, Hamamatsu University School of Medicine, 1-20-1, Handayama, Higashi-ku, Hamamatsu, Shizuoka, 431-3192, Japan

[2] Department of Orthopaedic Surgery, Kobe University Graduate School of Medicine, 7-5-1, Kusunoki-cho, Chuo-ku, Kobe, Hyogo, 650-0017, Japan

[3] Ushio Inc., Marunouchi Kitaguchi Building, 1-6-5, Marunouchi, Chiyoda-ku, Tokyo, 100-8150, Japan

[4] Department of Microbiology and Immunology, Hamamatsu University School of Medicine, 1-20-1, Handayama, Higashi-ku, Hamamatsu, Shizuoka, 431-3192, Japan

[5] Department of Perioperative Functioning Care and Support, Hamamatsu University School of Medicine, 1-20-1, Handayama, Higashi-ku, Hamamatsu, Shizuoka, 431-3192, Japan

*Corresponding author

E-mail: kurachi1@hama-med.ac.jp (KK)

Abstract

Far ultraviolet-C irradiation at a wavelength of 222 nm has strong bactericidal effects against severe infections, such as peritonitis, but is rarely cytotoxic. Bacterial peritonitis due to bowel perforation is a severe disease with a high mortality rate despite having available treatment options. The effects of intraperitoneal far ultraviolet-C irradiation at 222 nm are unknown; therefore, herein, we investigated its safety and efficacy. We performed *in vitro* experiments to optimize the fluid conditions of bacterial or protein concentration for the *in vivo* evaluation of the bactericidal effect. We evaluated the efficacy of far ultraviolet-C irradiation at 222 nm during lavage of Escherichia coli-induced peritonitis rats by analyses of the intra-abdominal bacteria concentrations, blood cytokine concentrations, and mortality. We also evaluated the safety of far ultraviolet-C irradiation at 222 nm by evaluation of cyclobutane pyrimidine dimers as DNA damage in five abdominal organs, namely the stomach, small intestine, colon, liver, and spleen. Statistical analyses were performed using parametric analysis in the data with normality, and using non-parametric analysis in the data without normality. The results of the *in vitro* experiments revealed the optimal conditions is, 10^6 CFU/mL of bacteria, 0.5 mW/cm^2 of irradiation, and 10^{-3} mg/mL of protein. In the rat peritonitis model, far ultraviolet-C irradiation at 222 nm reduced the intra-abdominal bacteria and blood cytokine (interleukin-1β and interleukin-6) concentrations and improved mortality (from 20% to 60%) compared with only lavage. Cyclobutane pyrimidine dimer formation was less observed in organ tissues with far ultraviolet-C irradiation at 222 nm than in those with ultraviolet-C irradiation at 254 nm. Our findings suggest far ultraviolet-C irradiation at 222 nm during lavage as a therapeutic against bacterial peritonitis, with a good safety profile.

Introduction

Acute peritonitis, caused by gastrointestinal perforation, surgical site infection, or spontaneous bacterial infection, is a severe disease with high morbidity and mortality. Despite available multimodal therapies, the 90-day mortality rate of acute diffuse peritonitis remains over 10% [1]. During laparotomy, resection of the infected area and intraperitoneal lavage with saline were performed to reduce bacterial concentration and control infection [2]. These surgical procedures and the following antibiotic administration are established treatments for acute peritonitis [2–9]. Although a large lavage decreases intra-abdominal bacterial concentration, it is difficult to completely eradicate the intra-abdominal infection [1,10]; therefore, novel treatments for intra-abdominal infections are required.

The strong bactericidal effect of ultraviolet-C (UV-C) irradiation has been well established. UV-C irradiation within the range of 200–280 nm has a strong bactericidal effect [11]. In particular, UV-C irradiation at 254 nm has a strong bactericidal effect and is often used in bactericidal equipment; however, it exhibits strong cytotoxicity through DNA damage, resulting in eye keratitis and malignant skin tumors [12–14], which hinders its clinical use.

Recently, far UV-C, defined as a wavelength of 200–230 nm, is attracting attention because it has an equal bactericidal effect and is safer for mammalian tissues than UV-C. The bactericidal effect of far UV-C irradiation at 222 nm has especially been reported. Indeed, far UV-C irradiation at 222 nm has been shown to decrease the concentration of methicillin-resistant *Staphylococcus aureus* on the skin surface and exert a bactericidal effect equivalent to or more than that of UV-C irradiation at 254 nm [15,16]. In addition, far UV-C irradiation at 222 nm is less cytotoxic to the skin, subcutaneous tissue, and eyes compared with UV-C irradiation at 254 nm [15,17,18].

A previous clinical trial showed that far UV-C irradiation at 222 nm of the skin at 500

mJ/cm^2 was safe for use in humans [19]. Furthermore, far UV-C irradiation at 222 nm has an equivalent bactericidal effect and less cytotoxicity compared with UV-C irradiation at 254 nm [16] and may therefore be a novel treatment method for intraperitoneal infection.

The bactericidal effects and cytotoxicity of UV-C irradiation depend on the relationship between the absorption coefficient and cell size [20]; therefore, they differ among bacterial species and cell types. Moreover, the bactericidal effect of UV-C irradiation is influenced by several factors, such as bacterial concentration, irradiance, fluid protein concentration, and transmittance rate [21–23]. Previous studies have focused on the therapeutic effect of far UV-C irradiation at 222 nm on intra-abdominal infections [15,16]; however, the effects of far UV-C irradiation at 222 nm on contaminated ascites containing bacteria and proteins remain unknown. The objective of this study was to evaluate the bactericidal effect and cytotoxicity of far UV-C irradiation at 222 nm in the intra-abdominal cavity both *in vitro* and *in vivo*.

Materials and methods

Experimental design

In vitro experiments were conducted to optimize the condition for the *in vivo* investigation of the bactericidal effect of far UV-C irradiation at 222 nm; the *in vivo* study comprised three experiments. First, we evaluated the efficacy of far UV-C irradiation at 222 nm for peritonitis using two protocols：acute phase (1 week) and hyper-acute phase (3 h) following the induction of peritonitis. Second, we evaluated the damage to the abdominal organs induced by far UV-C irradiation at 222 nm.

Study approval

All animal procedures and experimental protocols were performed in accordance with the ethical standards of the Animal Care and Use Committee of the Hamamatsu University School of Medicine (approval number 2022008). The study protocols complied with the Animal Research Reporting of In Vivo Experiments guidelines.

Bacterial culture and measurement

Escherichia coli (Migula) Castellani and Chalmers (ATCC 25922) was obtained from the American Type Culture Collection (Manassas, Virginia, US) and cultured in tryptic soy broth (BD #211825, BD Difco US Co., Ltd.) at 37 °C with SLI-1200 (Tokyo Rikakikai Co., Ltd., Tokyo Japan). To determine the bacterial concentration, the colony-forming units (CFU)/mL were measured after streak-plate culture and diluted 10–10^{10}-fold with sterilized saline. From each diluted solution, 100 μL was added to a deoxycholate-agar plate, streaked, and incubated for 24–48 h at 37 °C.

UV-C irradiation

A 222 nm-emitting Care222® device (Ushio Inc. Tokyo, Japan) was used for far UV-C irradiation at 222 nm, as previously described [18,20,24], and the irradiance emitted by the 222-nm light was measured with an S-172/UIT250 accumulated UV meter (Ushio Inc.). UV-C irradiation (254 nm) was performed using a low-pressure mercury lamp (SUV-4, AS ONE, Osaka, Japan). The irradiance of UV-C at 254 nm was measured similar to that of far UV-C at 222 nm.

The irradiance of far UV-C at 222 nm is inversely proportional to the square of the distance, that is, the longer the distance from the irradiation device, the lower the irradiance of far UV-C at 222 nm. Furthermore, areas with sufficient irradiance are small over long distances.

Therefore, we adjusted the irradiance and distance using a peritonitis rat model. The irradiance was 0.5 mW/cm^2 at 20 cm and 0.25 mW/cm^2 at 40 cm from this device. It was difficult to irradiate at > 0.5 mW/cm^2 using this device because the distance was too short between the light source and rat model or at < 0.25 mW/cm^2 because the area with sufficient irradiance was too small.

Bactericidal effect of far UV-C irradiation at 222 nm *in vitro*

On a clean bench, *E. coli* dilutions in sterile saline were dispensed into microwells at a depth of 1 cm. According to previously reported experimental protocols [25,26], the doses of far UV-C irradiation was divided into low dose (LD, 20 mJ/cm^2) and high dose (HD, 500 mJ/cm^2). The *E. coli* solution was exposed to far UV-C irradiation at 222 nm at the above two doses. The microwells were shaken gently during irradiation. After irradiation, the bacterial concentrations were measured and compared with those of the negative control (NC, not irradiated).

A reduction of > 2 log CFU/mL, which indicates > 99% reduction, was considered to denote a confirmed bactericidal effect [15]. We also confirmed the influence of irradiance on the bactericidal effect of far UV-C irradiation at 222 nm. The irradiance of far UV-C at 222 nm was set at 0.25 or 0.5 mW/cm^2. After irradiation, the bacterial concentrations were measured and compared among the three groups (NC, LD, and HD).

We evaluated the bactericidal effect of far UV-C irradiation at 222 nm in protein-containing solutions with soy broth as pseudo-ascites, performed multiple dilutions of soy broth with saline, and adjusted the protein concentration from 1.0×10^{-3} to 1.5 mg/mL. *E. coli* dilutions in soy broth solutions were also dispensed into microwells at a depth of 1 cm and exposed to far UV-C irradiation at 222 nm.

We evaluated the influence of protein levels and solution depth on the transmittance rate of far UV-C irradiation at 222 nm. The concentrations of soy broth solution were adjusted to 1.0 × 10^{-3}, 1.5 × 10^{-3}, and 1.5 × 10^{-2} mg/mL, while the irradiance was adjusted to 0.5 mW/cm^2 in blank using a luminometer placed on the bottom of the quartz cells. The protein solution was added to quartz cells at a depth of 1.5, 3.0, and 4.5 cm. We irradiated the quartz cuvettes with far UV-C at 222 nm, blocking the cuvettes from surrounding light. The transmittance rate was calculated using the ratio of far UV-C irradiance at 222 nm applied at a depth of 1.5, 3.0, and 4.5 cm compared with the blank quartz cuvettes (n = 5 in each soy broth concentration).

Rat peritonitis model

For the *in vivo* experiments, 16–18-week-old male Sprague-Dawley rats (Japan SLC, Inc., Hamamatsu, Japan; 450–500 g) were used. All rats were intraperitoneally anesthetized with 0.4 mg/kg medetomidine, 2.0 mg/kg midazolam, and 5.0 mg/kg butorphanol before the surgical procedures. The rats were divided into two groups: control and peritonitis (n = 5 per group). In the peritonitis group, 2 mL of *E. coli* solution adjusted to 10^8 CFU/mL with saline was injected into the lower right abdomen [27]. On the other hand, 2 mL of saline was injected in the control group. Three hours after injection, we measured the ascitic bacterial and serum cytokine concentrations and compared the control and peritonitis groups (Fig 1A).

Bactericidal effect of far UV-C irradiation on peritonitis

We used two protocols to evaluate the bactericidal effect of far UV-C irradiation at 222 nm on acute and hyper-acute peritonitis using a rat model. We performed laparotomy once in the acute phase protocol and twice in the hyper-acute phase protocol.

The evaluation protocol during the acute phase is illustrated in Fig 1B. Twenty

Sprague-Dawley rats were randomly divided into two groups (non-irradiated and irradiated, n = 10 in each group). *E. coli* solution was injected into all rats as described above. Three hours after the injection of *E. coli* solution, laparotomy was performed. In the non-irradiated group, intra-abdominal lavage was performed twice with 50 mL of saline; irradiation was not performed. Conversely, in the irradiated group, intra-abdominal lavage was performed twice with 50 mL of saline, and far UV-C irradiation at 222 nm at 20 mJ/cm^2 (0.5 mJ/cm^2 for 40 s) was performed during the second lavage. In both groups, 5 mL of a second lavage solution was obtained immediately before abdominal closure. The survival rate was evaluated, and the surviving rats were sacrificed with an overdose of intraperitoneal anesthetics (1.2 mg/kg medetomidine, 6.0 mg/kg midazolam, and 15.0 mg/kg butorphanol) 1 week after laparotomy to obtain the cecum and to evaluate the DNA damage induced by far UV-C irradiation using histopathological evaluation. The cecum is large and susceptible to irradiation; hence, it was included in the analysis.

Fig 1. *In Vivo* Experimental Protocols to Evaluate the Bactericidal Effect of Far UV-C Irradiation on Peritonitis. (A) Sample collection of rat peritonitis model. Ten rats were divided into control and peritonitis group (n = 5 in each). Three hours after the injection of *E. coli* solution or saline, the laparotomy was performed, and blood samples and ascites were collected. (B) Evaluation in the acute phase. Twenty rats were divided into non-irradiated and irradiated groups (n = 10 in each). Three hours after the injection of *E. coli* solution, laparotomy was performed. Lavage was performed in the non-irradiated group and lavage and irradiation, in the irradiated group. After these procedures, the lavage solution was collected. The survival rate was evaluated, and the surviving rats were sacrificed 1 week after the procedure to obtain the cecum. (C) Evaluation in the hyper-acute phase. Thirty rats were divided into peritonitis (n = 20) and sham groups (n = 10). Each group was divided into two sub-groups, namely non-

irradiated and irradiated. Three hours after the injection of *E. coli* solution or saline, initial laparotomy was performed. Lavage was performed in the non-irradiated group and lavage and irradiation, in the irradiated group. Three hours after the procedures, the second laparotomy was performed, and blood samples and ascites were collected.

The evaluation protocol for the hyper-acute phase is illustrated in Fig 1C. Thirty Sprague-Dawley rats were randomly divided into two groups, peritonitis (n = 20) and sham (n = 10). *E. coli* solution was injected into the rats in the peritonitis group, while saline was injected into those in the sham group. The rats in each group were divided into two sub-groups, namely irradiated and non-irradiated. Three hours after the injection of *E. coli* solution or saline, the initial laparotomy was performed. In the non-irradiated group, intra-abdominal lavage was performed twice with 50 mL of saline, while irradiation was not performed. In the irradiated group, intra-abdominal lavage was performed twice with 50 mL of saline, and far UV-C irradiation at 222 nm at 20 mJ/cm^2 (0.5 mJ/cm^2 for 40 s) was performed during the second lavage. The abdomen was closed after the procedure. Three hours after the initial laparotomy, a second one was performed. Blood samples were collected from the portal vein. The volume of the ascitic fluid was too small to be collected and analyzed. The lavage solution was collected after dispensing 10 mL of saline into the abdominal cavity and gently stirring.

Analysis of lavage solution and blood samples

The bacterial concentration (CFU/mL) in the lavage solution was determined after streak-plate culturing and diluted 10–10^{10}-fold with sterilized saline.

The protein concentration of the lavage solution was measured. The lavage solution was added into a quartz cuvette and measured with spectrophotometry using an analyzer (DS-

11+, Denovix Inc., Wilmington, DE, US) and the ProteinA280 software (Thermo Fisher Scientific Inc., Commonwealth of Massachusetts, US).

Within 30 min of collection, the blood samples were centrifuged at 3,000 rpm for 5 min, and serum was collected and stored frozen at -80 °C. Based on the datasheet, the interleukin (IL)-1β, IL-6, and tumor necrosis factor (TNF-α) concentrations were measured using the Luminex® Discovery Assay Rat Premixed Multi-Analyte Kit (R&D Systems, Inc. Minneapolis, MN, US). The analysis was performed by Filgen, Inc. (Nagoya, Japan) using a Bio-Plex (TM) 200 System (Bio-Rad Laboratories Inc., California, US), and the analysis software was Bio-Plex Manager (TM) Software Version 6.1 (Bio-Rad Laboratories, Inc.).

Evaluation of cytotoxicity in the abdominal tissues by UV-C irradiation

Twenty rats were randomly divided into four groups: NC, LD, HD, and positive control (PC) (n = 5 per group). Under anesthesia with similar doses at the induction of peritonitis, laparotomy was performed through a ventral midline abdominal incision. The abdominal tissues in the NC group were not exposed to UV-C irradiation. The irradiance was set at 0.5 mW/cm^2, and the abdominal tissues were exposed to far UV-C irradiation at 222 nm at 20 mJ/cm^2 (0.5 mJ/cm^2 for 40 s) in the LD group and 500 mJ/cm^2 (0.5 mJ/cm^2 for 1000 s) in the HD group. In the PC group, the irradiance was set at 0.5 mW/cm^2, and the abdominal tissues were exposed to UV-C irradiation at 254 nm at 75 mJ/cm^2 (0.5 mJ/cm^2 for 150 s). After irradiation, five tissue samples (small intestine, ascending colon, stomach, liver, and spleen) were obtained from each rat, fixed in formalin, and underwent histopathological analysis to evaluate UV-C induced DNA damage.

Immunohistochemical staining for cyclobutane pyrimidine dimers

Immunohistochemical (IHC) staining for cyclobutane pyrimidine dimers (CPD) was performed to evaluate UV-C induced DNA damage [20]. The obtained abdominal tissues were fixed in 4% formaldehyde at 24°C for 24 h and embedded in paraffin wax. The deparaffinized and rehydrated sliced sections (3 μm-thick) were incubated with CPD antibody (1:1,000, Cosmo Bio Co., Ltd., Tokyo, Japan), diluted with phosphate-buffered saline containing 1% bovine serum albumin, overnight at 4°C. Histofine Simple Stain MAX Po (M) (Nichirei Bioscience Inc., Tokyo, Japan) was used as a secondary antibody, with incubation for 40 minutes. Simple Stain aminoethyl carbazole solution (Nichirei Bioscience Inc.) was used for detection. The slides were counterstained with hematoxylin. Images were captured using a BX51 microscope (Olympus, Tokyo, Japan).

Three fields were randomly selected, and the CPD-positive rate and depth were measured. The numbers of CPD-positive and CPD-negative cells were counted. The percentage of CPD-positive cells indicated the CPD-positive rate [20], while the distance from the tissue surface to the deepest CPD-positive cells indicated the CPD-positive depth [28].

Statistical analyses

The distribution of continuous variables was assessed using the Kolmogorov–Smirnov test. Normality was confirmed for the transmittance rate, CPD-positive rate, and CPD-positive depth, and the data are shown as the mean and standard deviation. Differences between these data were analyzed using parametric analysis: Student's *t*-test between two groups or analysis of variance (ANOVA), followed by post hoc analysis with Tukey’s procedure, among more than two groups. Normality was not confirmed for the bacterial, protein, and serum cytokine (IL-1β, IL-6, and TNF-α) concentrations. Data are presented as the median and interquartile

range. Differences between these data were analyzed using non-parametric analysis: Mann–Whitney *U* test between two groups or Kruskal-Wallis test, followed by post hoc analysis with the Dunn-Bonferroni procedure, among more than two groups.

The survival rates between the irradiated and non-irradiated groups were compared using Kaplan–Meier analysis with the log-rank test. Correlations between the bacterial concentration and each blood cytokine level were analyzed using Pearson's correlation coefficients. A correlation was considered significant at $r > 0.4$ or < -0.4, and statistical significance was set at $p < 0.05$. The results were analyzed using SPSS version 28 (IBM Co., Ltd. New York, US).

Results

Bactericidal effect of far UV-C irradiation at 222 nm *in vitro*

Through *in vitro* experiments, we optimized the conditions for bacterial concentration, irradiance, and protein concentration for the *in vivo* experiments.

E. coli was diluted with saline to a concentration of $10^{5\text{-}7}$ CFU/mL. The irradiance of far UV-C at 222 nm was 0.5 mW/cm^2, and the NC, LD, and HD groups were compared. The bacterial concentration in the $10^{5–7}$ CFU/mL solution significantly decreased after far UV-C irradiation at 222 nm (bacterial concentration of 10^7 CFU/ml, NC: 2.0×10^7 CFU/mL [1.8×10^7–2.2×10^7], LD: 3.4×10^5 CFU/mL [2.0×10^5–1.2×10^6], HD: 2.5×10^4 CFU/mL [7.7×10^3–5.5×10^4], bacterial concentration of 10^6 CFU/ml, NC: 2.0×10^6 CFU/mL [1.8×10^6–2.2×10^6], LD: 1.6×10^2 CFU/mL [1.4×10^2–1.8×10^2], HD: 0 CFU/mL [0–5.0], bacterial concentration of 10^5 CFU/ml, NC: 2.0×10^5 CFU/mL [1.8×10^5–2.2×10^5], LD: 0 CFU/mL [0–9.0×10^1], HD: 0×0 CFU/mL [0–0], $p < 0.01$) (Fig 2A). The bacterial concentration in the $10^{5\text{-}7}$ CFU/mL solution significantly decreased in the LD and HD groups compared with the

NC group ($p < 0.01$). The bacterial concentrations in the 10^6 and 10^7 CFU/mL solutions were significantly different between the LD and HD groups ($p < 0.01$). The bacterial concentration in the 10^7 CFU/mL solution between the NC and LD groups and between the LD and HD groups showed a reduction of < 2 log CFU/mL. In the 10^6 CFU/ml solution, the bacterial concentrations between the NC and LD groups and between the LD and HD groups were reduced by > 2 log CFU/mL. In the 10^5 CFU/ml solution, the bacterial concentrations between the NC and LD groups were reduced by > 2 log CFU/mL, while no significant difference was observed between the LD and HD groups ($p = 0.690$). Differences of > 2 log CFU/mL between the NC and LD groups and between the LD and HD groups were only observed in the 10^6 CFU/mL solution.

Fig 2. Bactericidal Effect of Far UV-C Irradiation at 222 nm *In Vitro*. (A-C) Comparison of the bacterial concentration among the normal control (NC), low dose (LD), and high dose (HD) groups in different conditions. (A) Assessment of different bacterial concentrations: 1.0×10^7, 1.0×10^6, and 1.0×10^5 CFU/mL (n = 5 per group). (B) Assessment of different irradiance conditions: 0.5 and 0.25 mW/cm^2 (n = 5 per group). (C) Assessment of different protein concentrations. *E. coli* was diluted with saline or soy broth solution in concentrations of 1.5, 1.5×10^{-1}, and 1.5×10^{-2} mg/mL (n = 5 per group). (D) The transmittance rate of far UV-C irradiation at 222 nm in the protein-containing solution at different concentrations; 1.0×10^{-3}, 1.5×10^{-3}, and 1.5×10^{-2} mg/ml (n = 5 per group). $^{**}p < 0.01$ (post hoc analysis with Tukey's procedure).

The influence of irradiance on the bactericidal effect of far UV-C irradiation at 222 nm is shown in Fig 2B. *E. coli* was diluted in saline to a concentration of 10^6 CFU/mL. The bacterial concentration significantly decreased following exposure to far UV-C irradiation at

222 nm at both 0.25 and 0.5 mW/cm^2 (bacterial concentration of 0.5 mW/cm^2, 10^7 CFU/ml, NC: 2.0×10^7 CFU/mL [1.8×10^6–2.2×10^6], LD: 1.6×10^2 CFU/mL [1.4×10^2–1.8×10^2], HD: 0 CFU/mL [0–5.0], bacterial concentration of 0.25 mW/cm^2, NC: 2.0×10^6 CFU/mL [1.8×10^6–2.2×10^6], LD: 5.3×10^5 CFU/mL [3.9×10^5–7.5×10^5], HD: 1.4×10^5 CFU/mL [1.1×10^5–1.7×10^5], $p < 0.01$) (Fig 2B). The bacterial concentrations were significantly different between the NC and LD groups and between the LD and HD groups at both 0.25 and 0.5 mW/cm^2 ($p < 0.01$). At an irradiance of 0.5 mW/cm^2, the bacterial concentrations between the NC and LD groups and between the LD and HD groups were reduced by > 2 log CFU/mL, while at an irradiance of 0.25 mW/cm^2, a reduction of > 1 log CFU/mL was observed.

We evaluated the bactericidal effect of far UV-C irradiation at 222 nm on protein-containing solutions. *E. coli* was diluted to a concentration of 10^6 CFU/mL with saline and soy broth solution to adjust the concentration from 1.5×10^{-2} to 1.5 mg/mL. The irradiance of far UV-C at 222 nm was set at 0.5 mW/cm^2, and the NC, LD, and HD groups were compared (Fig 2C). At a soy broth concentration of 1.5 mg/mL, no significant difference among the NC, LD, and HD groups was observed. At a soy broth concentration of 1.5×10^{-1} and 1.5×10^{-2} mg/mL, the bacterial concentrations significantly differed among the three groups (bacterial concentration of saline, NC: 2.2×10^6 CFU/mL [1.9×10^6–2.4×10^6], LD: 1.6×10^2 CFU/mL [1.4×10^2–1.8×10^2], HD: 0 CFU/mL [0–5.0], bacterial concentration of 1.5 mg/ml, 10^7 CFU/ml, NC: 2.0×10^6 CFU/mL [1.9×10^6–2.4×10^6], LD: 1.9×10^6 CFU/mL [1.4×10^6–2.1×10^6], HD: 1.7×10^6 CFU/mL [1.4×10^6–2.1×10^6], bacterial concentration of 1.5×10^{-1} mg/ml, NC: 2.0×10^6 CFU/mL [1.9×10^6–2.4×10^6], LD: 2.3×10^5 CFU/mL [1.8×10^5–4.0×10^5], HD: 1.6×10^3 CFU/mL [1.2×10^3–7.8×10^3] , bacterial concentration of 1.5×10^{-2} mg/ml, NC: 2.0×10^6 CFU/mL [1.9×10^6–2.4×10^6], LD: 2.0×10^2 CFU/mL [1.4×10^2–4.0×10^2], HD: 0 CFU/mL [0–0], $p < 0.01$). At a soy broth concentration of 1.5×10^{-1} mg/mL, the bacterial concentrations showed a reduction of < 2 log CFU/mL between the NC and LD groups and

between the LD and HD groups, while at a concentration of 0 (saline) and 1.5×10^{-2} mg/mL, the bacterial concentrations were reduced by > 2 log CFU/mL between the NC and LD groups and between the LD and HD groups.

We evaluated the effect of protein concentration and solution depth on the transmittance rate of far UV-C irradiance at 222 nm (Fig 2D). The concentrations of the soy broth solution were adjusted to 1.0×10^{-3}, 1.5×10^{-3}, and 1.5×10^{-2} mg/mL. The irradiance of far UV-C at 222 nm was set at 0.5 mW/cm^2. The transmittance rate was significantly different among the different soy broth concentrations (transmittance rate of 1.5×10^{-2} mg/ml, 1.5 cm: 0.343 ± 0.068, 3.0 cm: 0.140 ± 0.025, 4.5 cm: 0.0522 ± 0.0067; transmittance rate of 1.5×10^{-3} mg/ml, 1.5 cm: 0.892 ± 0.046, 3.0 cm: 0.768 ± 0.080, 4.5 cm: 0.662 ± 0.116; transmittance rate of 1.0×10^{-3} mg/ml, 1.5 cm: 0.944 ± 0.008, 3.0 cm: 0.866 ± 0.010, 4.5 cm: 0.793 ± 0.022; $p < 0.01$). Significant differences in the transmittance rate were observed between concentrations of 1.0×10^{-3} and 1.5×10^{-2} mg/mL ($p < 0.01$) and those of 1.5×10^{-3} and 1.5×10^{-2} mg/mL ($p < 0.01$). At a depth of 4.5 cm, the transmittance rate was approximately 10% at a soy broth concentration of 1.5×10^{-2} mg/mL. The transmittance rate was > 50% and significantly increased at soy broth concentrations of 1.0×10^{-3} and 1.5×10^{-3} mg/mL compared with that at 1.5×10^{-2} mg/mL ($p < 0.01$). Based on these results, a bacterial concentration of 1.0×10^{6} CFU/mL, an irradiance of 0.5 mW/cm^2 of far UV-C at 222 nm, and a protein concentration of < 1.5×10^{-3} mg/mL were desirable for the *in vivo* experiments.

Evaluation of the rat peritonitis model

To confirm the induction of peritonitis in rats, bacteria in ascites and blood cytokines were evaluated 3 hours after *E. coli* administration. All rats in the control and peritonitis groups survived three hours after injection. The bacterial and serum cytokine concentration

significantly increased in the peritonitis group compared with the control group (Fig 3A-D) ((bacterial concentration, control: 0 CFU/mL [0–0], peritonitis group: 1.9×10^9 CFU/mL [3.4×10^7–4.8×10^9], $p < 0.01$), (IL-1β, control: 0 pg/mL [0–3.26], peritonitis group: 183.0 pg/mL [47.6–201.5], $p < 0.01$), (IL-6, control: 0 pg/mL [0–0], peritonitis group: 6649.3 pg/mL [2381.5–8168.1], $p < 0.01$), (TNF-α, control: 17.9 pg/mL [2.8–24.5], peritonitis group: 226.3 pg/mL [155.9–298.8], $p < 0.01$)).

Fig 3. Evaluation of Ascites and Serum Cytokine Levels in the Peritonitis Rat Model. (A) Bacterial concentration in ascites of the control and peritonitis groups (n = 5 per group). The bacterial concentration significantly increased in the peritonitis group. (B-D) Serum cytokine levels of the control and peritonitis groups (n = 5 per group). (B) interleukin (IL)-1β, (C) IL-6, and (D) tumor necrosis factor (TNF-α). The serum cytokine levels significantly increased in the peritonitis group. **$p < 0.01$ (Student's *t*-test).

Bactericidal effect of far UV-C irradiation on peritonitis in the acute phase

In a rat peritonitis model, we evaluated survival rate and DNA damage for the organ in the irradiation area as the effects of far UV-C irradiation at 222nm UV-C during lavage. The 1-week survival rate was significantly higher in the irradiated groups than in the non-irradiated group ($p = 0.038$, 60% vs. 20%) (Fig 4A). The bacterial concentration in the lavage solution was significantly decreased in the irradiated group than in the non-irradiated group (irradiated: 1.5×10^3 CFU/mL [2.0×10^2–7.9×10^4], non-irradiated: 2.5×10^5 CFU/mL [1.7×10^5–2.3×10^6], $p < 0.01$) (Fig 4B). The protein concentrations in the lavage solution were $< 1.5 \times 10^{-3}$ mg/mL in both groups, with no significant difference between them (data not shown). Both the

bacterial and protein concentrations met the conditions for the *in vitro* experiments.

Fig 4. Evaluation of the Bactericidal Effect of Far UV-C Irradiation on the Rat Peritonitis Model in the Acute Phase. (A) Survival rate during the 7 days after each procedure in the irradiated and non-irradiated groups (n = 10 per group, $p = 0.038$, log-rank test). (B) Bacterial concentration in ascites of the non-irradiated and irradiated groups (n = 10 per group). $^{**}p < 0.01$ (Mann–Whitney *U* test). (C) Cyclobutane pyrimidine dimer (CPD)-positive ratio and (D) depth in the cecum obtained on day 7 (non-irradiated, n = 2; irradiated group: n = 6).

The cecum was obtained from the surviving rats on day 7; of the surviving rats, two were in the non-irradiated group and six in the irradiated group. The CPD-positive rate was 0.21% ± 0.068% in the irradiated group and 0.19% ± 0.067% in the non-irradiated group ($p = 0.636$) (Fig 4C). The CPD-positive depth was 4.34 ± 0.64 μm in the irradiated and 4.37 ± 0.70 μm in the non-irradiated groups ($p = 0.920$) (Fig 4D). There were no significant differences in the CPD-positive rate or depth.

Bactericidal effect of far UV-C irradiation on peritonitis in the hyper-acute phase

In vivo study at hyper-acute phase was conducted to evaluate the influence of far UV-C irradiation to blood cytokine level and bacterial concentration in ascites. In the peritonitis group, the bacterial concentrations significantly decreased in the irradiated group compared with the non-irradiated group (irradiated: 4.1×10^2 CFU/mL [1.3×10^2–1.1×10^3], non-irradiated: 1.3×10^5 CFU/mL [1.2×10^4–5.8×10^5], $p < 0.01$) (Fig 5A).

The serum IL-1β and IL-6 levels significantly decreased in the irradiated peritonitis

group compared with the non-irradiated group (IL-1β; irradiated: 68.7 pg/mL [40.4–87.6], non-irradiated: 109.5 pg/mL [92.4–149.2], $p < 0.01$; IL-6; irradiated: 1,367.6 pg/mL [751.0–1,731.0], non-irradiated: 3458.2 pg/mL [2,374.3–4,973.8], $p = 0.01$) (Fig 5B, C). In the sham group, no significant differences in the bacterial concentration and serum IL-1β and IL-6 levels between the irradiated and non-irradiated groups were observed (bacterial concentration; irradiated: 0 CFU/mL [0–0], non-irradiated: 0 CFU/mL [0–0], $p = 1.000$) (IL-1β; irradiated: 0 pg/mL [0–4.4], non-irradiated: 0 pg/mL [0–11.1], $p = 0.841$) (IL-6; irradiated: 0 pg/mL [0–0], non-irradiated: 0 pg/mL [0–55.6], $p = 0.690$) (Fig 5A-C). Further, both in the sham and peritonitis groups, there were no significant differences in the serum TNF-α levels between the irradiated and non-irradiated groups (peritonitis group; irradiated: 73.3 pg/mL [58.9–146.3], non-irradiated: 103.0 pg/mL [69.7–194.8], $p = 0.436$; sham group; irradiated: 17.9 pg/mL [5.5–31.1], non-irradiated: 31.1 pg/mL [21.2–31.1], $p = 0.310$) (Fig 5D).

Fig 5. Evaluation of the Bactericidal Effect of Far UV-C Irradiation for the Peritonitis Rat Model in the Hyper-Acute Phase. Bacterial concentration in ascites (A) and serum interleukin (IL)-1β (B), IL-6 (C), and tumor necrosis factor (TNF-α) (D) levels in the sham (non-irradiated, n = 5, irradiated, n = 5) and peritonitis groups (non-irradiated, n = 10, irradiated, n = 10). The bacterial concentration and serum IL-1β and IL-6 levels significantly decreased in the irradiated peritonitis group (**$p < 0.01$, Student's *t*-test). Correlations between the bacterial concentration in ascites and serum IL-1β level (E) ($r = 0.644$, $p < 0.01$) and that between the bacterial concentration in ascites and serum IL-6 level (F) ($r = 0.838$, $p < 0.01$) in the peritonitis group. Gray circles indicate the irradiated group, and white circles indicate the non-irradiated group.

The relationship between the bacterial concentration and serum IL-1β and IL-6 levels

is shown in Fig 5E and 5F; a positive correlation was noted (bacterial concentration and serum IL-1β level; $r = 0.644$, 95% confidence interval [CI], 0.266–0.841, $p < 0.01$; bacterial concentration and serum IL-6 level; $r = 0.838$, 95% CI, 0.615–0.931, $p < 0.01$).

DNA damage in the abdominal tissues by far UV-C irradiation

We evaluated DNA damage to intraperitoneal organs by far UV-C irradiation to confirm the safety of this procedure. IHC for CPDs was performed to evaluate the DNA damage induced by UV-C irradiation (Fig 6A). CPD-positive cells were not observed in any tissue in the NC group, while only a few CPD-positive cells were observed in the serosa of abdominal tissues in the LD and HD groups. In the PC group, many CPD-positive cells were observed in the serosa and inside tissues. The CPD-positive rate was significantly lower for all tissues in the LD and HD groups than for those in the PC groups (CPD-positive rates of the small intestine, NC: 0% ± 0, LD: 0.92% ± 0.31, HD: 1.10% ± 0.48, PC: 6.97% ± 1.90, CPD-positive rates of the colon, NC: 0% ± 0, LD: 0.83% ± 0.28, HD: 1.27% ± 0.39, PC: 10.99% ± 2.17, CPD-positive rates of the stomach, NC: 0% ± 0, LD: 0.75% ± 0.16, HD: 1.24% ± 0.28, PC: 22.27% ± 5.45, CPD-positive rates of the liver, NC: 0% ± 0, LD: 2.44% ± 1.00, HD: 3.84% ± 0.54, PC: 11.55% ± 2.83, CPD-positive rates of the spleen, NC: 0% ± 0, LD: 0.45% ± 0.10, HD: 1.74% ± 0.22, PC: 12.00% ± 1.97) (Fig 6B). There was no significant difference in the CPD-positive rate in the small intestine, ascending colon, stomach, or spleen between the LD and NC groups or between the HD and NC groups. There is a significant difference in the CPD-positive rate in the liver alone between the LD and NC groups and between the HD and NC groups.

The CPD-positive depth was significantly lower in all tissues in the LD and HD groups than in those in the PC group and significantly higher in all tissues except for the spleen in the LD than in the NC group (between the LD and NC groups) (CPD-positive depths of the small

intestine, NC: 0 μm ± 0, LD: 4.31 μm ± 1.47, HD: 5.44 μm ± 1.78, PC: 24.92 μm ± 1.36, CPD-positive depths of the colon, NC: 0 μm ± 0, LD 5.05 μm ± 1.36, HD: 5.67 μm ± 1.59, PC: 60.28 μm ± 3.25, CPD-positive depths of the stomach, NC: 0 μm ± 0, LD 5.69 μm ± 1.76, HD: 6.06 μm ± 1.54, PC: 61.03 μm ± 6.38, CPD-positive depths of the liver, NC: 0 μm ± 0, LD: 4.34 μm ± 0.42, HD: 5.15 μm ± 1.05, PC 31.45% ± 1.05, CPD-positive depths of the spleen: NC: 0 μm ± 0, LD: 2.80 μm ± 0.94, HD: 4.13 μm ± 1.07, PC: 36.21 μm ± 2.28.) (Fig 6C).

Fig 6. Cytotoxicity in the Abdominal Tissues by UV-C Irradiation. (A) Immunohistochemical (IHC) staining for cyclobutane pyrimidine dimers (CPDs) in the irradiated abdominal tissues (NC, negative control; LD, low dose; HD, high dose; PC, positive control). Arrowheads indicate CPD-positive cells. Scale bars = 20 μm. CPD-positive rate (B) and CPD-positive depth (C) of the irradiated abdominal tissues (n = 5 per group). $^{**}p < 0.01$ and $^{*}p < 0.05$ compared with the NC group, $^{\dagger\dagger}p < 0.01$ compared with the PC group, $^{\ddagger}p < 0.05$, ANOVA followed by post hoc analysis with Tukey’s procedure.

Discussion

Lavage removes visible contamination and helps dilute bacteria and other biochemical substances against gastrointestinal peritonitis [9]. Several attempts have been made to add antibiotics or disinfectants during lavage to enhance surgical treatment, although the results remain inconsistent [9,29–35]. Several studies have shown that intraperitoneal lavage with antibiotics and disinfectants exacerbates systemic inflammation in peritonitis [9,36–38].

The appropriate lavage volume remains to be determined in both humans and animals. Since a large volume of saline lavage alone cannot reduce postoperative mortality and complications of acute peritonitis with gastrointestinal perforation, we developed an approach

for intra-operative bacterial reduction. Recent studies have found that far UV-C irradiation at 222 nm exerts a strong bactericidal effect on various sites [15,16]. This study reports the efficacy of far UV-C irradiation at 222 nm against bacterial peritonitis.

In this study, one critical issue was that the clinical ascites with peritonitis contained proteins and other biochemical substances. Specifically, far UV-C irradiance at 222 nm is strongly absorbed by proteins; hence, its ability to penetrate the clinical ascites after peritonitis is limited compared with that of an experimental saline solution [24,25]. Therefore, identifying and optimizing appropriate irradiation conditions were necessary to approximate our experimental model to clinical conditions. We demonstrated that a strong bactericidal effect could be obtained with far UV-C irradiance at 222 nm even in a diluted protein-containing solution. However, high *E. coli* concentration in the *in vitro* experiments masked the actual sensitivity of the microorganism.

E. coli densities of > 10^7 CFU/mL may promote cell aggregation [39]. Lavage with agitation could lead to bacterial liberation and optimize the effect of far UV-C irradiation at 222 nm; far UV-C irradiance at 222 nm cannot reach aggregated *E. coli* but can attack liberated *E. coli* by lavage. In fact, far UV-C irradiation at 222 nm showed a weak bactericidal effect, even with insufficient dilution and irradiance, which was the result of an attack on *E. coli* liberated on the surface of the solution. Based on these *in vitro* results, we imitated the clinical conditions with achievable bacterial concentrations by lavage in the first step of our *in vivo* studies and evaluated the safety of far UV-C irradiation at 222 nm to the intra-abdominal organs.

The core concept of this study was whether the bacterial reduction in ascites improved systemic inflammation and survival in bacterial peritonitis. A previous study reported bacterial concentrations of < 10^6 CFU/mL in 50% of cases after lavage during elective colorectal surgery, even without visible contamination [10]. These bacteria cause postoperative infections in proportion to the increase in bacterial levels. In bacterial peritonitis, persistent infection by

ascites without visible contamination can be a major challenge. Our experimental model mimicked severe peritonitis without any visible contamination after lavage. Short-term far UV-C irradiation at 222 nm induced a significant bacterial reduction beyond lavage and improved systemic inflammation and survival. This confirms the importance of immediate bacterial reduction in the treatment of severe bacterial peritonitis, even if bacterial contamination is undetectable after lavage.

The association between bacterial reduction, systemic inflammation, and mortality in *E. coli*-induced peritonitis was assessed in this study. In the irradiated group of rat peritonitis model, the IL-1β and IL-6 blood levels, and the bacterial concentration in lavage solution were significantly reduced, and the survival rate was improved. High IL-1β and IL-6 levels are associated with a high mortality risk of bacterial peritonitis [40-42]. Far UV-C irradiation at 222 nm strongly inhibited the secretion of these pro-inflammatory cytokines with a bactericidal effect, demonstrating its potential to treat peritonitis by impeding the release of pro-inflammatory cytokines.

Confirming the safety of far UV-C irradiation at 222 nm to the intra-abdominal organs was another challenge in this study. To the best of our knowledge, studies on the effects of UV-C irradiation on the intra-abdominal organs, including that at 222 nm, are lacking. UV-C irradiation at both 222 and 254 nm has a strong bactericidal effect. In hospital settings, UV-C light systems can be used to reduce the spread of pathogens. These bactericidal effects are attributed to DNA damage, such as CPD formation [21]. DNA damage represses transcription and replication and induces cell death. UV-C irradiation at 222 nm is more easily absorbed by proteins in the cytoplasm than that at 254 nm [21].

This high absorption results in < 1 µm of 50% intracellular transmission of irradiation at 222 nm, as opposed to 3 µm of transmission of that at 254 nm [22,23]. The diameters of mammalian cells are 10–25 µm [43], while those of bacteria are < 1 µm [43,44]; therefore, far

UV-C irradiation at 222 nm can easily reach the nucleus of bacterial cells compared with that of mammalian cells. Herein, we have demonstrated that far UV-C irradiation at 222 nm to the intra-abdominal organs can be safely performed without cell damage, compared with UV-C irradiation at 254 nm.

Based on previous findings, we presumed that far UV-C irradiation at 222 nm exerts bactericidal effects against *E. coli* by damaging its DNA [45]. However, the photoinactivation mechanism of far UV-C irradiation at 222 nm remains unclear. Lipopolysaccharide (LPS) is a component of the outer membrane of gram-negative bacteria that plays a role in its pathogenesis. LPS triggers a strong immune response in the host, inducing the release of pro-inflammatory cytokines and contributing to the symptoms associated with bacterial infections [40-42]. While the direct relationship between the LPS activation pathway and far UV-C irradiation at 222 nm remains unclear, they have several implications in various fields such as medicine, environmental health, and biotechnology [46–50]. Therefore, we intend to investigate the relationship between far UV-C irradiation at 222 nm and the LPS-induced inflammation pathway in future studies.

In this study, we demonstrated the efficacy of far UV-C irradiation at 222 nm in an *E. coli*-induced peritonitis model. In previous studies, far UV-C irradiation at 222 nm has been shown to induce a bactericidal effect against various bacteria, as well as some viruses and fungi [15,16,45,51]. Antimicrobial-resistant bacteria have hindered the treatment of infectious diseases globally [52]. Further, delayed treatment of antibiotic-resistant bacteria can lead to fatal consequences in bacterial peritonitis. Intra-operative far UV-C irradiation at 222 nm can exert therapeutic effects against various pathogenic microorganisms, including antimicrobial-resistant bacteria. In future studies, we intend to determine whether UV-C irradiation at 222 nm induces a bactericidal effect on various bacteria in ascites.

The 222-nm far UV-C device has been commercialized and widely used; besides

equipment installation, this device requires no additional costs for irradiation. Moreover, far UV-C irradiation at 222 nm is safe and effective in various mammalian infection sites; therefore, a far UV-C irradiation device at 222 nm can be used in conjunction with lighting equipment in operating rooms.

Conclusion

This study showed that, at the appropriate bacterial and protein concentrations and irradiance, far UV-C intraperitoneal irradiation at 222 nm exerted sufficient bactericidal effects both *in vivo* and *in vitro*. DNA damage in the abdominal organs was also acceptable at an irradiance of 0.5 mW/cm^2. Therefore, far UV-C irradiation at 222 nm is a potentially effective treatment for peritonitis, and further research for clinical application is required.

Acknowledgments

The authors would like to thank Editage (www.editage.jp) for English language editing.

Author contributions

Conceptualization: Kosuke Sugiyama, Kiyotaka Kurachi, Hiroya Takeuchi

Data curation: Kosuke Sugiyama, Kiyotaka Kurachi, Tomoaki Fukui, Toru Koi

Formal analysis: Kosuke Sugiyama, Kiyotaka Kurachi, Masaki Sano, Kyota Tatsuta, Tadahiro Kojima, Toshiya Akai, Katsunori Suzuki, Kakeru Torii, Mayu Sakata, Yoshifumi Morita, Hirotoshi Kikuchi, Yoshihiro Hiramatsu, Hiroya Takeuchi

Funding acquisition: Mayu Sakata, Kiyotaka Kurachi, Hiroya Takeuchi

Investigation: Kosuke Sugiyama

Methodology: Kosuke Sugiyama, Kiyotaka Kurachi, Masaki Sano, Kyota Tatsuta, Tadahiro Kojima, Toshiya Akai, Katsunori Suzuki, Kakeru Torii, Mayu Sakata, Yoshifumi Morita, Hirotoshi Kikuchi, Yoshihiro Hiramatsu, Tomoaki Fukui, Rena Kaigome, Masahiro Sasaki, Toru Koi, Tetsuro Suzuki, Ryosuke Kuroda, Hiroya Takeuchi

Resources: Rena Kaigome, Masahiro Sasaki, Toru Koi, Hiroyuki Ohashi, Tetsuro Suzuki,

Supervision: Toru Koi, Ryosuke Kuroda, Hiroya Takeuchi

Validation: Rena Kaigome, Masahiro Sasaki, Toru Koi, Hiroyuki Ohashi

Writing – original draft: Kosuke Sugiyama, Masaki Sano

Writing – review & editing: Kosuke Sugiyama, Kiyotaka Kurachi, Masaki Sano, Kyota Tatsuta, Tadahiro Kojima, Toshiya Akai, Katsunori Suzuki, Kakeru Torii, Mayu Sakata, Yoshifumi Morita, Hirotoshi Kikuchi, Yoshihiro Hiramatsu, Yohei Kumabe, Keisuke Oe, Tomoaki Fukui, Rena Kaigome, Masahiro Sasaki, Toru Koi, Hiroyuki Ohashi, Tetsuro Suzuki, Ryosuke Kuroda, Hiroya Takeuchi

Data availability

The data that support the findings of this study are available from the corresponding author, KK, upon reasonable request.

References

1. Marubashi S, Takahashi A, Kakeji Y, Hasegawa H, Ueno H, Eguchi S, et al. Surgical outcomes in gastroenterological surgery in Japan: report of the National Clinical Database 2011-2019. Ann Gastroenterol Surg. 2021;5: 639-658. doi: 10.1002/ags3.12462

2. Binda GA, Papa A, Persiani R, Escalante R, De Oliveira EC, Crucitti A, et al. Hot topics in surgical management of acute diverticulitiss. J Gastrointestin Liver Dis. 2019;28: 29-34. doi: 10.15403/jgld-555

3. Schein M, Gecelter G, Freinkel W, Gerding H, Becker PJ. Peritoneal lavage in abdominal sepsis. A controlled clinical study. Arch Surg. 1990;125: 1132-1135. doi: 10.1001/archsurg.1990.01410210058008

4. Hau T, Ahrenholz DH, Simmons RL. Secondary bacterial peritonitis: the biologic basis of treatment. Curr Probl Surg. 1979;16: 1-65. doi: 10.1016/s0011-3840(79)80011-8

5. Biondo S, Jaurrieta E, Jorba R, Moreno P, Farran L, Borobia F, et al. Intraoperative colonic lavage and primary anastomosis in peritonitis and obstruction. Br J Surg. 1997;84: 222-225. doi: 10.1046/j.1365-2168.1997.02497.x

6. Vennix S, Musters GD, Mulder IM, Swank HA, Consten EC, Belgers EH, et al. Laparoscopic peritoneal lavage or sigmoidectomy for perforated diverticulitis with purulent peritonitis: a multicentre, parallel-group, randomised, open-label trial. Lancet. 2015;386: 1269-1277. doi: 10.1016/S0140-6736(15)61168-0

7. Thornell A, Angenete E, Bisgaard T, Bock D, Burcharth J, Heath J, et al. Laparoscopic lavage for perforated diverticulitis with purulent peritonitis: a randomized trial. Ann Intern Med. 2016;164: 137-145. doi: 10.7326/M15-1210

8. Kohl A, Rosenberg J, Bock D, Bisgaard T, Skullman S, Thornell A, et al. Two-year

results of the randomized clinical trial DILALA comparing laparoscopic lavage with resection as treatment for perforated diverticulitis. Br J Surg. 2018;105: 1128-1134. doi: 10.1002/bjs.10839

9. Rosman C, Westerveld GJ, Kooi K, Bleichrodt RP. Local treatment of generalised peritonitis in rats; effects on bacteria, endotoxin and mortality. Eur J Surg. 1999;165: 1072-1079. doi: 10.1080/110241599750007928

10. Claesson BE, Holmlund DE. Dip slide culture of intraoperative peritoneal irrigation fluid for prediction of septic complication in elective colorectal surgery. J Clin Microbiol. 1986;24: 922-928. doi: 10.1128/jcm.24.6.922-928.1986

11. Bintsis T, Litopoulou-Tzanetaki E, Robinson RK. Existing and potential applications of ultraviolet light in the food industry - a critical review. J Sci Food Agric. 2000;80: 637-645. doi: 10.1002/(SICI)1097-0010(20000501)80:6<637::AID-JSFA603>3.0.CO;2-1

12. Sliney D. Balancing the risk of eye irritation from UV-C with infection from bioaerosols. Photochem Photobiol. 2013;89: 770-776. doi: 10.1111/php.12093

13. Sterenborg HJ, van der Putte SC, van der Leun JC. The dose-response relationship of tumorigenesis by ultraviolet radiation of 254 nm. Photochem Photobiol. 1988;47: 245-253. doi: 10.1111/j.1751-1097.1988.tb02722.x

14. Kowalczuk CI, Priestner MC, Pearson AJ, Saunders RD, Bouffler SD. Wavelength dependence of cellular responses in human melanocytes and melanoma cells following exposure to ultraviolet radiation. Int J Radiat Biol. 2006;82: 781-792. doi: 10.1080/09553000600930111

15. Narita K, Asano K, Morimoto Y, Igarashi T, Hamblin MR, Dai T, et al. Disinfection and healing effects of 222-nm UVC light on methicillin-resistant Staphylococcus aureus infection in mouse wounds. J Photochem Photobiol B. 2018;178: 10-18. doi:

10.1016/j.jphotobiol.2017.10.030

16. Ponnaiya B, Buonanno M, Welch D, Shuryak I, Randers-Pehrson G, Brenner DJ. Far-UVC light prevents MRSA infection of superficial wounds in vivo. PLOS ONE. 2018;13: e0192053. doi: 10.1371/journal.pone.0192053

17. Yamano N, Kunisada M, Kaidzu S, Sugihara K, Nishiaki-Sawada A, Ohashi H, et al. Long-term effects of 222-nm ultraviolet radiation C sterilizing lamps on mice susceptible to ultraviolet radiation. Photochem Photobiol. 2020;96: 853-862. doi: 10.1111/php.13269

18. Fukui T, Niikura T, Oda T, Kumabe Y, Ohashi H, Sasaki M, et al. Exploratory clinical trial on the safety and bactericidal effect of 222-nm ultraviolet C irradiation in healthy humans. PLOS ONE. 2020;15: e0235948. doi: 10.1371/journal.pone.0235948

19. Narita K, Asano K, Morimoto Y, Igarashi T, Nakane A. Chronic irradiation with 222-nm UVC light induces neither DNA damage nor epidermal lesions in mouse skin, even at high doses. PLOS ONE. 2018;13: e0201259. doi: 10.1371/journal.pone.0201259

20. Fukui T, Niikura T, Oda T, Kumabe Y, Nishiaki A, Kaigome R, et al. Safety of 222 nm UVC irradiation to the surgical site in a rabbit model. Photochem Photobiol. 2022;98: 1365-1371. doi: 10.1111/php.13620

21. Buonanno M, Randers-Pehrson G, Bigelow AW, Trivedi S, Lowy FD, Spotnitz HM, et al. 207-nm UV light – a promising tool for safe low-cost reduction of surgical site infections. I: in vitro studies. PLOS ONE. 2013;8: e76968. doi: 10.1371/journal.pone.0076968

22. Coohill TP. Virus-cell interactions as probes for vacuum-ultraviolet radiation damage and repair. Photochem Photobiol. 1986;44: 359-363. doi: 10.1111/j.1751-1097.1986.tb04676.x

23. Green H, Boll J, Parrish JA, Kochevar IE, Oseroff AR. Cytotoxicity and mutagenicity

of low intensity, 248 and 193 nm excimer laser radiation in mammalian cells. Cancer Res. 1987;47: 410-413.

24. Goh JC, Fisher D, Hing ECH, Hanjing L, Lin YY, Lim J, et al. Disinfection capabilities of a 222 nm wavelength ultraviolet lighting device: a pilot study. J Wound Care. 2021;30: 96-104. doi: 10.12968/jowc.2021.30.2.96

25. Buonanno M, Welch D, Shuryak I, Brenner DJ. Far-UVC light (222 nm) efficiently and safely inactivates airborne human coronaviruses. Sci Rep. 2020;10: 10285. doi: 10.1038/s41598-020-67211-2.

26. Eadie E, Hiwar W, Fletcher L, Tidswell E, O'Mahoney P, Buonanno M, et al. Far-UVC (222 nm) efficiently inactivates an airborne pathogen in a room-sized chamber. Sci Rep. 2022;12: 4373. doi: 10.1038/s41598-022-08462-z.

27. Sánchez E, Such J, Chiva MT, Soriano G, Llovet T, Mercè J, et al. Development of an experimental model of induced bacterial peritonitis in cirrhotic rats with or without ascites. Am J Gastroenterol. 2007;102: 1230-1236. doi: 10.1111/j.1572-0241.2007.01182.x

28. Kaidzu S, Sugihara K, Sasaki M, Nishiaki A, Ohashi H, Igarashi T, et al. Re-evaluation of rat corneal damage by short-wavelength UV revealed extremely less hazardous property of far-UV-C[†]. Photochem Photobiol. 2021;97: 505-516. doi: 10.1111/php.13419

29. Lally KP, Shorr LD, Nichols RL. Aminoglycoside peritoneal lavage: lack of efficacy in experimental fecal peritonitis. J Pediatr Surg. 1985;20: 541-542. doi: 10.1016/s0022-3468(85)80484-x

30. Hau T, Jacobs DE, Hawkins NL. Antibiotics fail to prevent abscess formation secondary to bacteria trapped in fibrin clots. Arch Surg. 1986;121: 163-168. doi: 10.1001/archsurg.1986.01400020049005

31. Edmiston CE, Jr., Goheen MP, Kornhall S, Jones FE, Condon RE. Fecal peritonitis: microbial adherence to serosal mesothelium and resistance to peritoneal lavage. World J Surg. 1990;14: 176-183. doi: 10.1007/BF01664870

32. Rosato EF, Oram-Smith JC, Mullis WF, Rosato FE. Peritoneal lavage treatment in experimental peritonitis. Ann Surg. 1972;175: 384-387. doi: 10.1097/00000658-197203000-00010

33. Ericsson CD, Duke JH, Jr., Pickering LK. Clinical pharmacology of intravenous and intraperitoneal aminoglycoside antibiotics in the prevention of wound infections. Ann Surg. 1978;188: 66-70. doi: 10.1097/00000658-197807000-00011

34. Perdue PW, Kazarian KK, Nevola J, Law WR, Williams T. The use of local and systemic antibiotics in rat fecal peritonitis. J Surg Res. 1994;57: 360-365. doi: 10.1006/jsre.1994.1155

35. Simopoulos C, Kouskoukis C, Polychronides A, Bezirtzoglou E. Effect of different combinations of antibiotics on experimental septic peritonitis in rabbits. Int J Clin Lab Res. 1994;24: 167-170. doi: 10.1007/BF02592448

36. Cohen J, McConnell JS. Antibiotic-induced endotoxin release. Lancet. 1985;2: 1069-1070. doi: 10.1016/s0140-6736(85)90939-0

37. Araujo ID, Grossi GC, Diniz SO, Nunes TA, Braga EA, Cardoso VN. Effects of the povidone-iodine (PVPI) in treatment of bacterial peritonitis induced in rats. Acta Cir Bras. 2010;25: 322-327. doi: 10.1590/s0102-86502010000400004

38. Song HL, Zhang DM, Wen H, Wang M, Zhao N, Gao YH, et al. Peritoneal lavage with povidone-iodine solution in colorectal cancer-induced rats. J Surg Res. 2018;228: 93-99. doi: 10.1016/j.jss.2018.02.055

39. Zhukova LV, Kiwi J, Nikandrov VV. TiO2 nanoparticles suppress Escherichia coli cell division in the absence of UV irradiation in acidic conditions. Colloids Surf B

Biointerfaces. 2012;97: 240-247. doi: 10.1016/j.colsurfb.2012.03.010

40. Ohlsson K, Björk P, Bergenfeldt M, Hageman R, Thompson RC. Interleukin-1 receptor antagonist reduces mortality from endotoxin shock. Nature. 1990;348: 550-552. doi: 10.1038/348550a0

41. Yassad A, Husson A, Bion A, Lavoinne A. Synthesis of interleukin 1beta and interleukin 6 by stimulated rat peritoneal macrophages: modulation by glutamine. Cytokine. 2000;12: 1288-1291. doi: 10.1006/cyto.1999.0729

42. Payen D, Dupuis C, Deckert V, Pais de Barros JP, Rérole AL, Lukaszewicz AC, et al. Endotoxin mass concentration in plasma is associated with mortality in a multicentric cohort of peritonitis-induced shock. Front Med (Lausanne). 2021;8: 749405. doi: 10.3389/fmed.2021.749405

43. Buonanno M, Ponnaiya B, Welch D, Stanislauskas M, Randers-Pehrson G, Smilenov L, et al. Germicidal efficacy and mammalian skin safety of 222-nm UV light. Radiat Res. 2017;187: 483-491. doi: 10.1667/RR0010CC.1

44. Lorian V, Zak O, Suter J, Bruecher C. Staphylococci, in vitro and in vivo. Diagn Microbiol Infect Dis. 1985;3: 433-444. doi: 10.1016/0732-8893(85)90082-3

45. Narita K, Asano K, Naito K, Ohashi H, Sasaki M, Morimoto Y, et al. 222-nm UVC inactivates a wide spectrum of microbial pathogens. J Hosp Infect. 2020. doi: 10.1016/j.jhin.2020.03.030

46. Ha JW, Lee JI, Kang DH. Application of a 222-nm krypton-chlorine excilamp to control foodborne pathogens on sliced cheese surfaces and characterization of the bactericidal mechanisms. Int J Food Microbiol. 2017;243: 96-102. doi: 10.1016/j.ijfoodmicro.2016.12.005.

47. Schneier M, Razdan S, Miller AM, Briceno ME, Barua S. Current technologies to endotoxin detection and removal for biopharmaceutical purification. Biotechnol

Bioeng. 2020;117: 2588-2609. doi: 10.1002/bit.27362.

48. Gorman A, Golovanov AP. Lipopolysaccharide structure and the phenomenon of low endotoxin recovery. Eur J Pharm Biopharm. 2022;180: 289-307. doi: 10.1016/j.ejpb.2022.10.006.

49. Ning P, Han Y, Liu Y, Liu S, Sun Z, Wang X, et al. Study on disinfection effect of a 222-nm UVC excimer lamp on object surface. AMB Express. 2023;13: 102. doi: 10.1186/s13568-023-01611-1.

50. Wang Y, Ma B, He C, Xia D, Yin R. Nitrate protects microorganisms and promotes formation of toxic nitrogenous byproducts during water disinfection by far-UVC radiation. Environ Sci Technol. 2023;57: 9064-9074. doi: 10.1021/acs.est.3c00824.

51. Fukuda S, Nishikawa J, Kobayashi Y, Okabe Y, Fujii T, Kubota N, et al. The bactericidal effect of far-UVC on ESBL-producing Escherichia coli. Am J Infect Control. 2022. doi: 10.1016/j.ajic.2022.04.012

52. Huemer M, Mairpady Shambat S, Brugger SD, Zinkernagel AS. Antibiotic resistance and persistence-Implications for human health and treatment perspectives. EMBO Rep. 2020;21: e51034. doi: 10.15252/embr.202051034

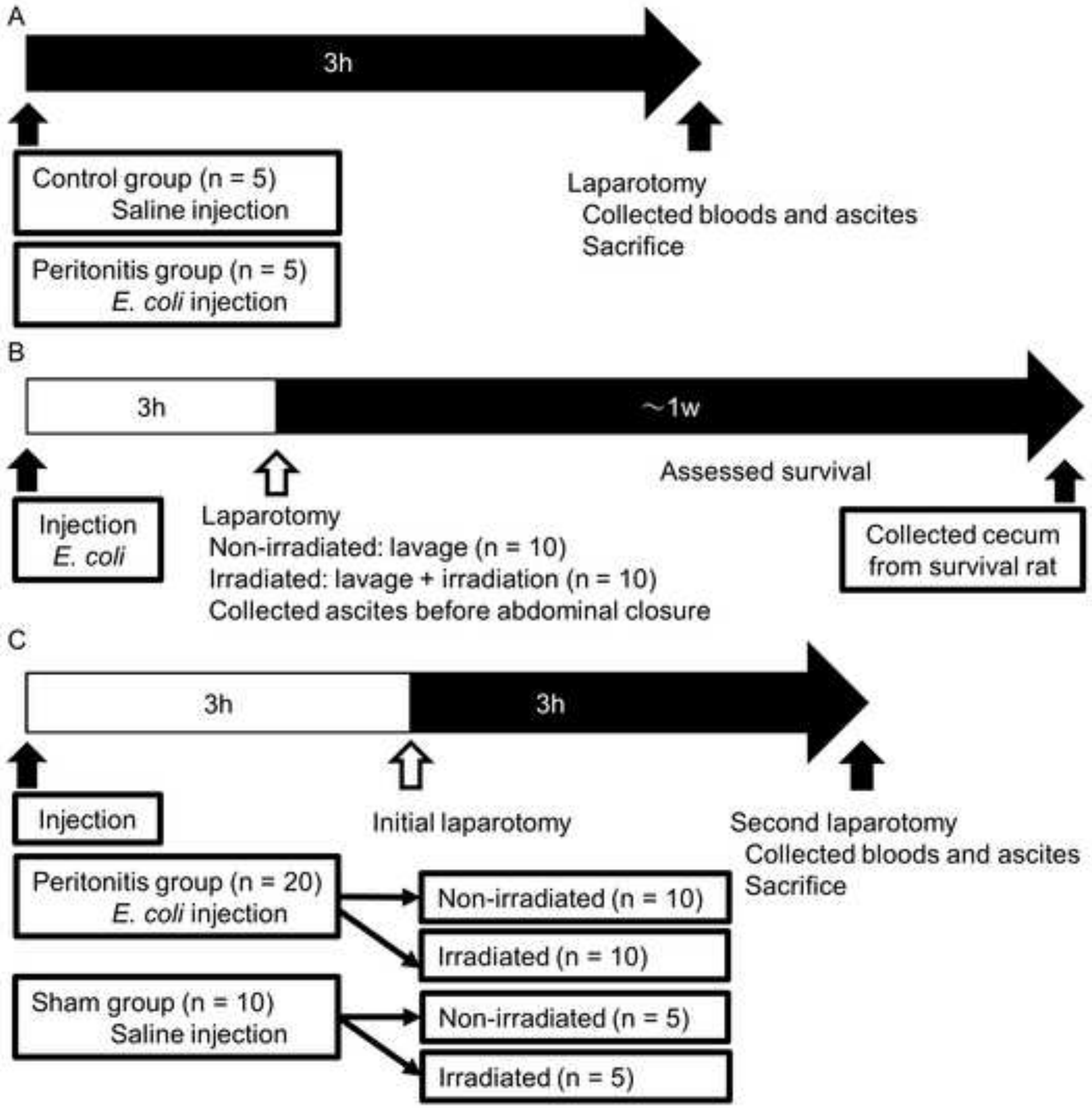
A
3h
Control group (n = 5)
Saline injection
Peritonitis group (n = 5)
E. coli injection
Laparotomy
Collected bloods and ascites
Sacrifice
B
3h
~1w
Injection
E. coli
Laparotomy
Non-irradiated: lavage (n = 10)
Irradiated: lavage + irradiation (n = 10)
Collected ascites before abdominal closure
Assessed survival
Collected cecum
from survival rat
C
3h
3h
Injection
Initial laparotomy
Second laparotomy
Collected bloods and ascites
Sacrifice
Peritonitis group (n = 20)
E. coli injection
Sham group (n = 10)
Saline injection
Non-irradiated (n = 10)
Irradiated (n = 10)
Non-irradiated (n = 5)
Irradiated (n = 5)

Figure2

Click here to access/download;Figure;Figure2.tif

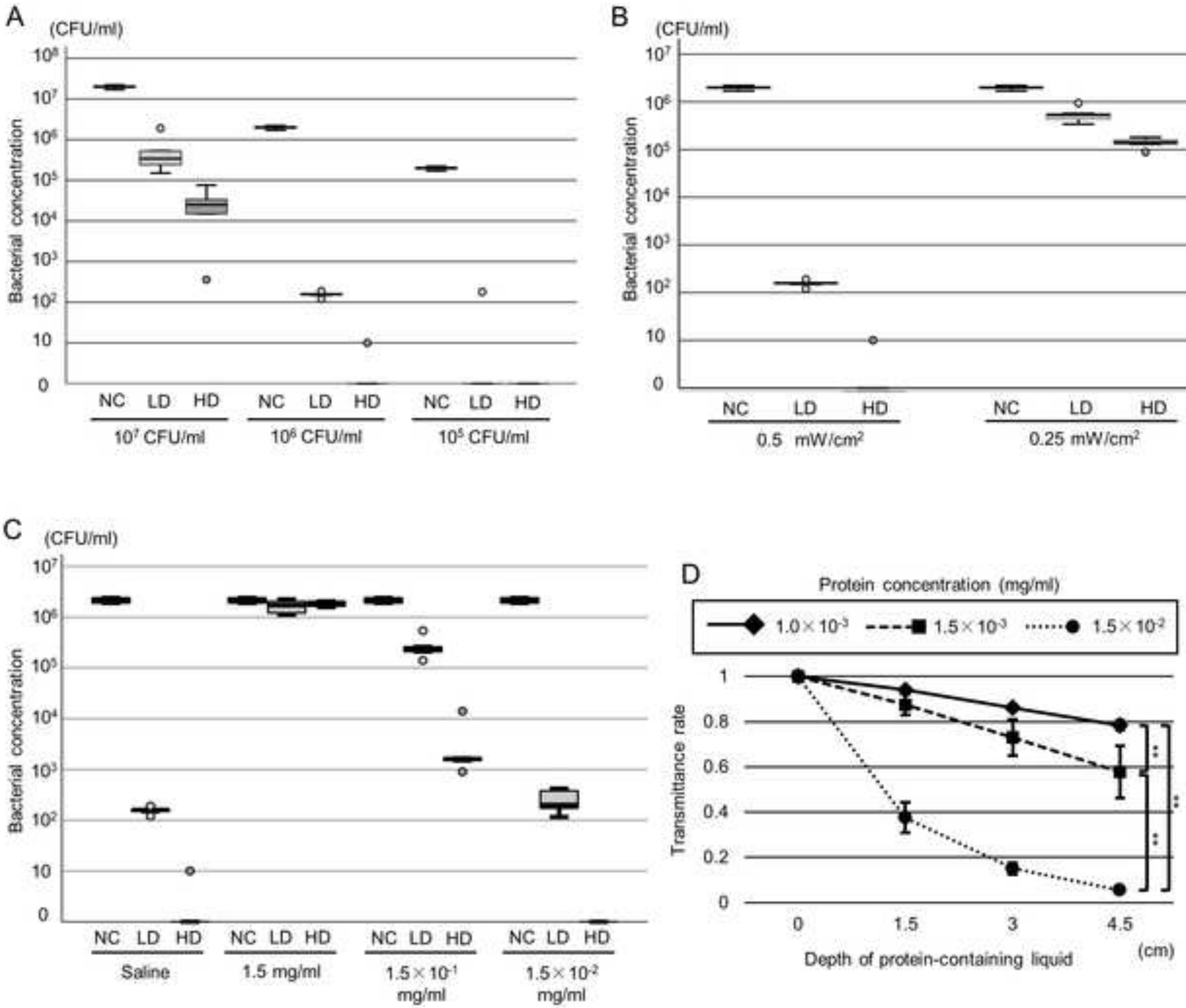

Figure3

Click here to access/download;Figure;Figure3.tif

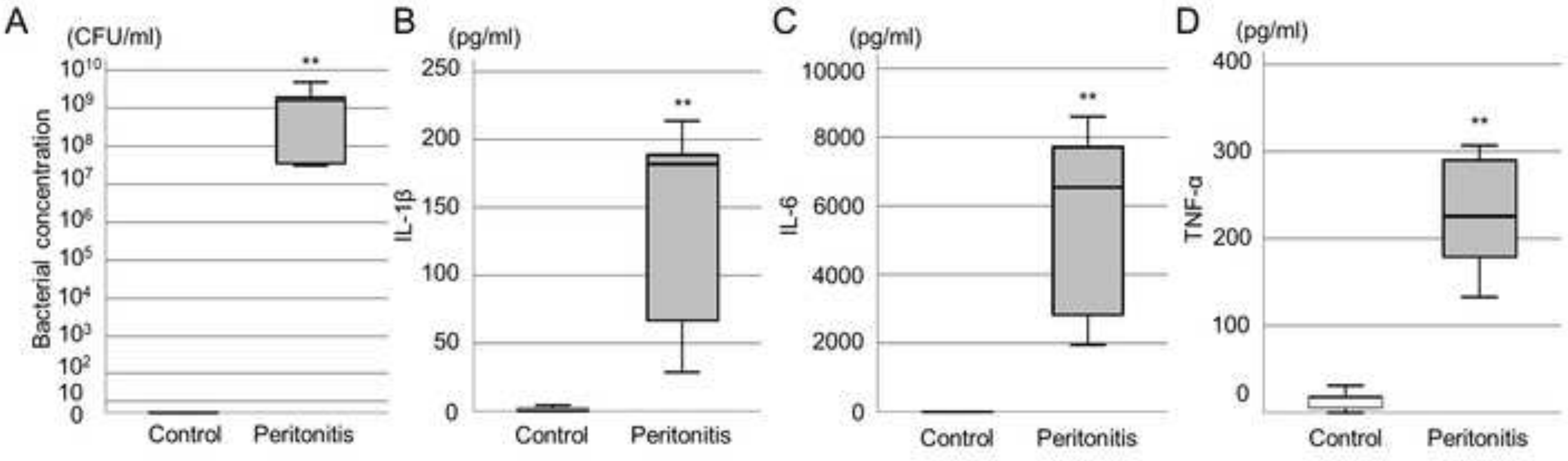

Figure4

Click here to access/download;Figure;Figure4.tif

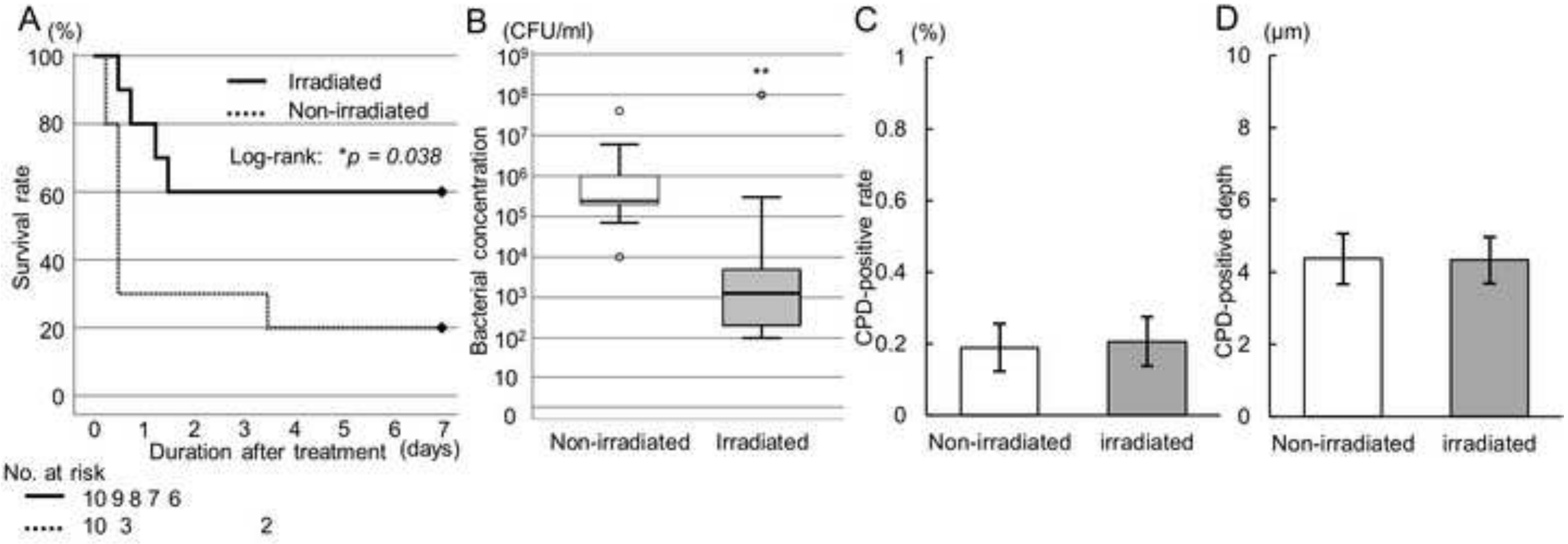

Click here to access/download;Figure;Figure5.tif

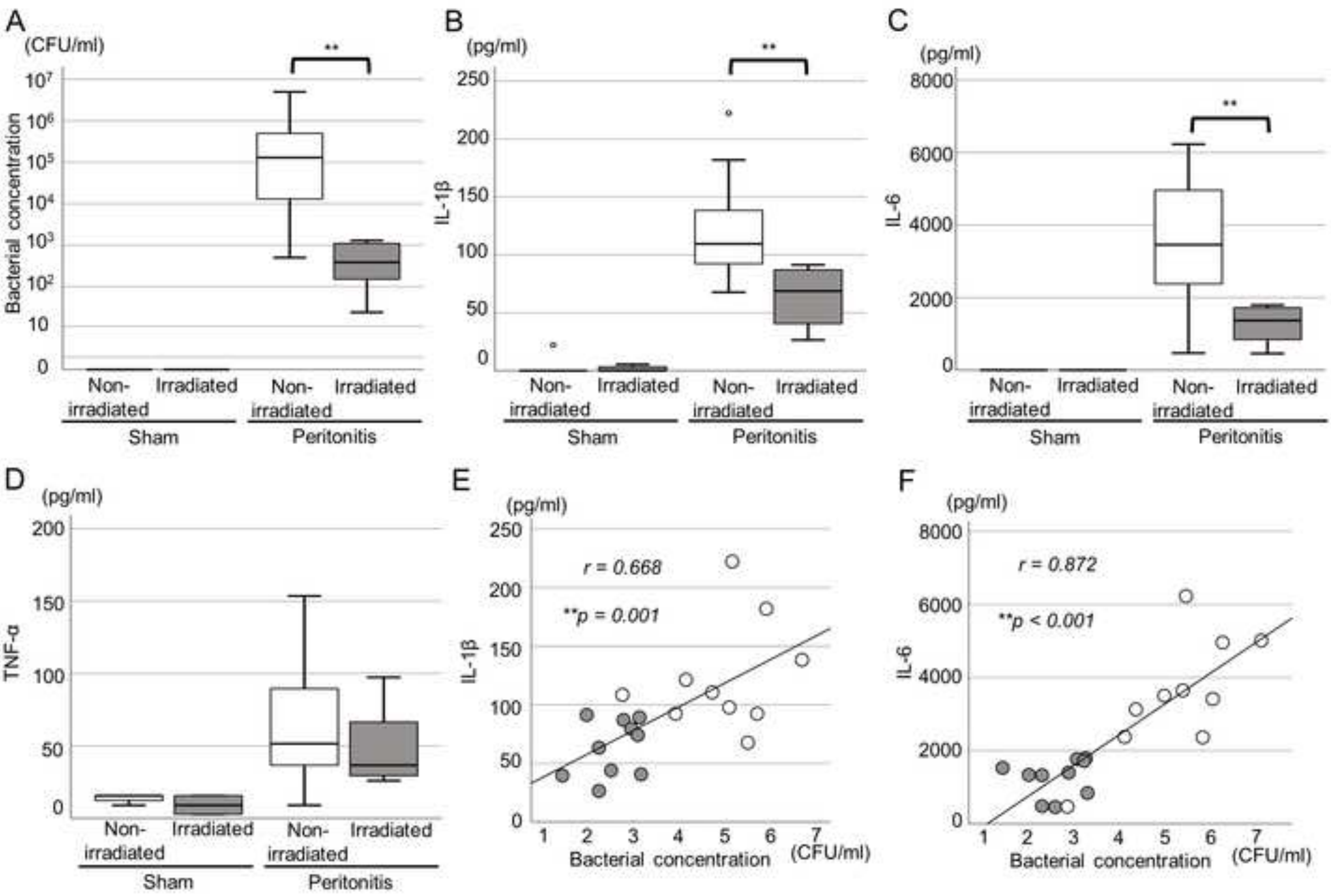

Figure6

Click here to access/download;Figure;Figure6.tif

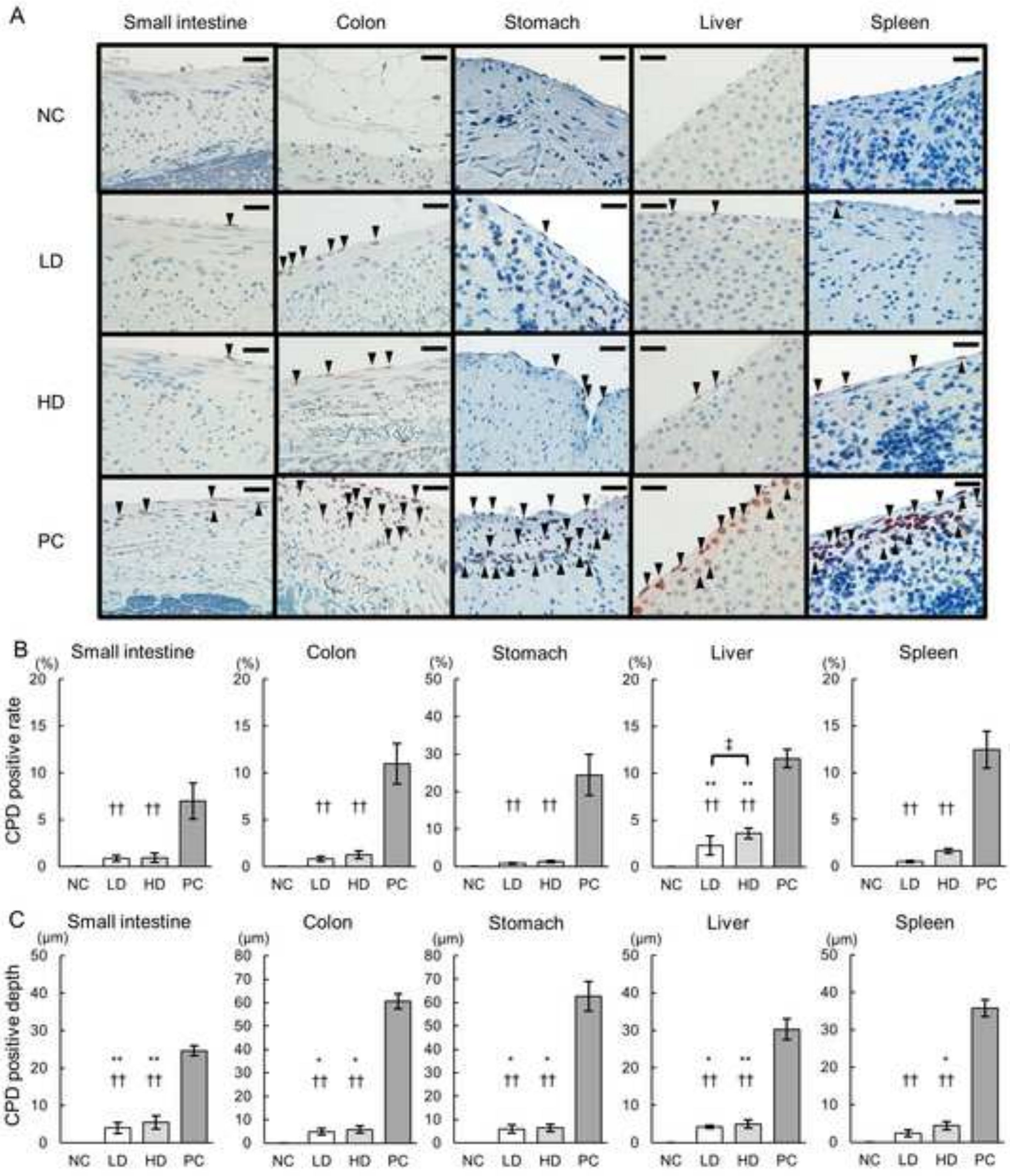

Supplement: Attachment — Submitted filename: PONE-D-24-00293_reviewer.pdf [file pone.0311552.s001.pdf]
