# Supplementary material for: Bactericidal effect of far ultraviolet-C irradiation at 222 nm against bacterial peritonitis
Source: PLoS One. 2024 Nov 12;19(11):e0311552. doi: 10.1371/journal.pone.0311552 (PMC11556692; doi:10.1371/journal.pone.0311552)
Supplement: Attachment — Submitted filename: PONE-D-24-0093.docx [file pone.0311552.s002.docx]

Comments

1- The text of the article has many problems in terms of writing and grammar.Edite the article in terms of grammar and writing.
2- What type of laser was used?
3- Explain the reason for using the wavelength of 222 and the time of irradiation?
4- There is no explanation about the distance from the laser to the peritonitis?
5- Why are variable wavelengths not used?
6- Why was antibiotics not used as a control?
