# Supplementary material for: Bactericidal effect of far ultraviolet-C irradiation at 222 nm against bacterial peritonitis
Source: PLoS One. 2024 Nov 12;19(11):e0311552. doi: 10.1371/journal.pone.0311552 (PMC11556692; doi:10.1371/journal.pone.0311552)
Supplement: Attachment — Submitted filename: Response to Reviewers.docx [file pone.0311552.s003.docx]

**Response to Reviewers**

Reviewer #1:

1. Please write the name of bacteria in italic format and apply it in the whole text.

Response: Thank you for your comments. I have italicized the name of the bacteria as per your suggestion. This adjustment appears in the abstract, specifically on lines 31 to 32 of page 2.

2. What was the reason for selecting this bacteria?

The selection of *Escherichia coli (E. coli)* in this research is critical due to its prevalence in severe colorectal perforation cases. *E. coli* not only triggers systemic inflammation through cytokine release but also secretes endotoxins. Therefore, assessing the impact of far UV-C irradiation at 222 nm on *E. coli* represents an essential preliminary step. We aim to extend this investigation to additional bacterial species in subsequent studies. We have incorporated the following into the Methods section on lines 118 to 120 of pages 5 to 6:

*"Escherichia coli (E. coli)* was selected due to its significant role in colorectal perforation and its ability to induce systemic inflammation through cytokine release and endotoxin production."

3. What about the time of irradation?

Response: As you indicated, the relationship between time, irradiance, and irradiation dose was not clear in the manuscript. It was essential to maintain a consistent irradiation dose in this study. I have included a formula to compute this relationship in the "Materials and Methods" section and further elucidated how irradiation time and irradiance influence the irradiation dose. This explanation is detailed on lines 138 to 146 of pages 6 to 7:

"The irradiation dose is calculated based on irradiance and exposure time, using the formula below. We categorized the irradiation dose as either low or high, based on the threshold limit values for 222 nm UV-C.

Irradiation dose ([mJ]⁄[cm^2^]) = Irradiance (mW⁄cm^2^) × Time (s)

For this study, consistent irradiation doses were required. Following the aforementioned formula, we adjusted the irradiance and distance in a peritonitis rat model. The settings were 0.5 mW/cm^2^ at a 20 cm distance for 40 s, and 0.25 mW/cm^2^ at a 40 cm distance for 1000 s."

4. Please write about the time too

Response: As demonstrated in the aforementioned formula, irradiation time varies with changes in irradiance. Importantly, we have documented the corresponding irradiation times for each irradiance level in the manuscript. The following has been added to the Methods section:

"According to previously reported experimental protocols [36,37], the doses of far UV-C irradiation were divided into low dose (LD, 20 mJ/cm^2^, 0.5 mW/cm^2^, 40 s) and high dose (HD, 500 mJ/cm^2^, 0.5 mW/cm^2^, 1000 s)." (lines 151 to 153 of page 7)

"The irradiance of far UV-C at 222 nm was set at 0.25 (LD: 80 sec, HD: 2000 sec) or 0.5 mW/cm^2^ (LD: 40 sec, HD: 1000 sec)." (lines 159 to 160 of page 7)

5. How did you confirm peritonitis? What about the acute one?

Response: Thank you for your observation. Confirming the onset of peritonitis is indeed crucial. Rats manifest behavioral changes such as reluctance to stretch the abdomen and immobility when they develop peritonitis. These behaviors were not present in preliminary experiments when the bacterial concentration was insufficient or the injection method was not intraperitoneal. Therefore, we confirmed the onset of peritonitis by monitoring these behavioral indicators and by measuring bacterial concentrations in the ascites and blood cytokine levels. We have included the following text in the manuscript on lines 185 to 188 of page 8:

“As physical findings at the time of peritonitis induction, rats with peritonitis tend to exhibit behavioral changes such as reluctance to stretch the abdomen and immobility. Additionally, as objective indicators to evaluate peritonitis, we measured the bacterial concentration in the ascites and blood cytokines to confirm their increase."

6. What about the time and safety of irradation? Please discuss about it.

Response: Thank you for your inquiry regarding the timing and safety of irradiation. The safety of 222 nm UV-C irradiation was evaluated by assessing CPD-positive ratio and depth. The study presented in Figure 1B shows no significant differences in CPD-positive ratio or depth between the irradiated and non-irradiated groups, suggesting that exposure to 222 nm UVC at a low dose (20 mJ/cm², 0.5 mW/cm², 40 sec) can be considered safe in the short term. Furthermore, in Figure 6, when comparing low and high doses of 222 nm UVC exposure, a significant increase in CPD-positive ratio was observed only at higher doses in liver tissues, indicating a dose-dependent increase in cytotoxicity. However, the CPD-positive depth remained below 10 µm in both scenarios, with no significant differences in CPD-positive depth noted, and no CPD-positive cells detected in the internal tissues of any organs in either group. These findings suggest that while the damage at high doses is confined to the organ surface, it does not guarantee long-term safety. Consequently, long-term follow-up studies are imperative to assess the enduring safety of 222 nm UV-C irradiation. We have updated the following text in the manuscript:

“CPD-positive cells were not observed in any tissue in the NC group, while only a few CPD-positive cells were observed in the serosa of abdominal tissues in the LD and HD groups. Conversely, there were no CPD-positive cells in the internal tissues.” (lines 479 to 482 of page 21)

“In the subsequent phase, we explored the safety of 222 nm UV-C irradiation in vivo. No difference in CPD-positive ratio or CPD-positive depth was observed between the group exposed to a low dose of 222 nm UV-C and the non-irradiated group. Consequently, exposure to 222 nm UV-C at a low dose appears to be safe in the short term. When comparing low and high doses of 222 nm UV-C exposure, the CPD-positive ratio increased significantly only at high doses in liver tissues, demonstrating a dose-dependent increase in cytotoxicity. However, the CPD-positive depth remained below 10 µm in both groups, with no significant difference noted. No CPD-positive cells were detected in the internal tissues of any organs in either group. These findings indicate that in the high-dose group, which was exposed for up to 1000 s, the damage is confined to the organ serosa, suggesting that the internal tissues remained undamaged. However, damage confined to the serosa does not guarantee long-term safety. Thus, prolonged follow-up studies are essential to assess the long-term safety of 222 nm UV-C irradiation.” (lines 573 to 585 of pages 24 to 25)

Reviewer #2:

1. The text of the article has many problems in terms of writing and grammar. Edit the article in terms of grammar and writing.

Response: Thank you for your feedback. We have meticulously reviewed the entire manuscript and implemented modifications to enhance its readability. Additionally, I have commissioned another round of English proofreading from Editage. The resubmitted manuscript has undergone further proofreading by Editage.

2. What type of laser was used?

We employed a KrCl excimer lamp and an irradiator equipped with a filter that excluded the UVC region, similar to devices used in prior studies [29, 31, 35]. We have added the following commentary to the Materials and Methods section on lines 130 to 132 of page 6.

"This device is a filtered KrCl excimer far UV-C, constructed with Krypton-Chloride (KrCl) excimer lamps and an irradiator with a filter that removes the UV-C region."

3. Explain the reason for using the wavelength of 222 and the time of irradiation?

Response: The rationale for selecting 222 nm UV-C includes its documented effectiveness and safety for treating superficial infections. The irradiation device is commercially available and widely accessible. Longer wavelengths of UV-C, such as 254 nm, are more cytotoxic, while shorter wavelengths can produce ozone and free radicals, leading to biological damage. We have added the following commentary to the Introduction section on lines 76 to 86 of page 4.

"Longer wavelengths of UV-C, such as 254 nm, have higher cytotoxicity, while shorter wavelengths can form ozone and free radicals, causing biological damage [25]. Recently, far UV-C, defined as wavelengths between 200–230 nm, has garnered interest for its comparable bactericidal effect and increased safety for mammalian tissues relative to traditional UV-C. Our study particularly concentrated on far UV-C irradiation at 222 nm, as devices for this wavelength are commercially available and easily accessible. Furthermore, far UV-C at 222 nm offers advantages with its effective bactericidal properties against skin surface infections and its reduced cytotoxicity to the skin, subcutaneous tissue, and eyes compared to UV-C at 254 nm [26-29]. Although devices with variable wavelengths are available, our focus was specifically on 222 nm due to its well-documented efficacy and safety in treating superficial infections [26-29]."

4. There is no explanation about the distance from the laser to the peritonitis?

Response: Concerning the irradiation distance, we have detailed this parameter in various sections of the manuscript. In this study, the irradiation doses for both the high-dose and low-dose groups were determined by the following formula: Irradiation dose (mJ/cm^2^) = Irradiance (mW/cm^2^) × Irradiation time (s). This explanation is detailed on lines 138 to 146 of pages 6-7:

"The irradiation dose is calculated based on irradiance and exposure time, using the formula below. We categorized the irradiation dose as either low or high, based on the threshold limit values for 222 nm UV-C.

Irradiation dose ([mJ]⁄[cm^2^]) = Irradiance (mW⁄cm^2^) × Time (s)

For this study, consistent irradiation doses were required. Following the aforementioned formula, we adjusted the irradiance and distance in a peritonitis rat model. The settings were 0.5 mW/cm^2^ at a 20 cm distance for 40 s, and 0.25 mW/cm^2^ at a 40 cm distance for 1000 s."

5. Why are variable wavelengths not used?

Response: While devices with variable wavelengths exist, we focused specifically on 222 nm due to its extensive research backing its bactericidal effectiveness and safety for superficial infections. In this research, we aimed to evaluate its efficacy on deeper infections, particularly peritonitis. We have added the following commentary to the Introduction section on lines 84 to 86 of page 4.

“Although devices with variable wavelengths are available, our focus was specifically on 222 nm due to its well-documented efficacy and safety in treating superficial infections [26-29].”

6. Why was antibiotics not used as a control?

Response: As noted, antibiotics are typically employed in treating peritonitis. To ascertain whether the bactericidal effect was attributable to UVC, lavage, or antibiotics, antibiotics were not used in this study. Instead, we assessed the supplementary effect of 222 nm UV-C irradiation, using peritoneal lavage as the control during surgical procedures.

We have added the following comments to the Methods section on lines 241 to 243 of page 11.

"In this study, antibiotics were not administered in any of the protocols. However, antibiotic administration is the established treatment for acute peritonitis. This may modify and obscure the therapeutic effects of intra-abdominal lavage and UVC treatment."
